# Mapping the landscape of HPV integration and characterising virus and host genome interactions in HPV‐positive oropharyngeal squamous cell carcinoma

**DOI:** 10.1002/ctm2.1556

**Published:** 2024-01-27

**Authors:** Shengming Xu, Chaoji Shi, Rong Zhou, Yong Han, NianNian Li, Chuxiang Qu, Ronghui Xia, Chunye Zhang, Yuhua Hu, Zhen Tian, Shuli Liu, Lizhen Wang, Jiang Li, Zhiyuan Zhang

**Affiliations:** ^1^ Department of Oral and Maxillofacial‐Head Neck Oncology Shanghai Ninth People's Hospital Shanghai Jiao Tong University School of Medicine Shanghai China; ^2^ College of Stomatology Shanghai Jiao Tong University Shanghai China; ^3^ National Center for Stomatology Shanghai China; ^4^ National Clinical Research Center for Oral Diseases Shanghai China; ^5^ Shanghai Key Laboratory of Stomatology, Shanghai Research Institute of Stomatology Shanghai China; ^6^ Research Unit of Oral and Maxillofacial Regenerative Medicine Chinese Academy of Medical Sciences Shanghai China; ^7^ Department of Bioinfomatics Sequanta Shanghai China; ^8^ Department of Oral Pathology Shanghai Ninth People's Hospital Shanghai Jiao Tong University School of Medicine Shanghai China

**Keywords:** host genomic alterations, HPV, oropharyngeal squamous cell carcinoma, viral integration

## Abstract

**Background:**

Human papillomavirus (HPV) integration into the host genome is an important factor in HPV(+)OPSCC carcinogenesis, in conjunction with HPV oncoproteins E6/E7. However, a well‐studied investigation about virus–host interaction still needs to be completed. Our objective is to characterise HPV integration to investigate potential mechanisms of tumourigenesis independent of E6/E7 oncoproteins.

**Materials and methods:**

High‐throughput viral integration detection was performed on 109 HPV(+)OPSCC tumours with relevant clinicopathological information. Of these tumours, 38 tumours underwent targeted gene sequencing, 29 underwent whole exome sequencing and 26 underwent RNA sequencing.

**Results:**

HPV integration was detected in 94% of tumours (with a mean integration count of 337). Tumours occurring at the tonsil/oropharyngeal wall that exhibit higher PD‐L1 expression demonstrated increased integration sites (*p* = .024). HPV exhibited a propensity for integration at genomic sites located within specific fragile sites (FRA19A) or genes associated with functional roles such as cell proliferation and differentiation (PTEN, AR), immune evasion (CD274) and glycoprotein biosynthesis process (FUT8). The viral oncogenes E2, E4, E6 and E7 tended to remain intact. HPV fragments displayed enrichment within host copy number variation (CNV) regions. However, insertions into genes related to altered homologous recombination repair were infrequent. Genes with integration had distinct expression levels. Fifty‐nine genes whose expression level was affected by viral integration were identified, for example, EPHB1, which was reported to be involved in cellular protein metabolic process.

**Conclusions:**

HPV can promote oncogenesis through recurrent integration into functional host genome regions, leading to subsequent genomic aberrations and gene expression disruption. This study characterises viral integrations and virus–host interactions, enhancing our understanding of HPV‐related carcinogenesis mechanisms.

## Graphical abstract

Viral breaking and integrating into the host genome were characterised, and integration hot spot genes were identified.

We characterised virus–host interactions and identified genes with co‐occurrence of CNVs or dysregulated expression and HPV integration.

Our observation supports viral integration exerts effects on genomic aberrations and host gene expression disruption, facilitate the shaping of distinct molecular features and tumour microenvironment of HPV(+)OPSCC.

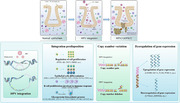

## 1 INTRODUCTION

Oropharyngeal squamous cell carcinoma (OPSCC) is classified into two distinct subtypes: Human papillomavirus (HPV)‐positive and HPV‐negative. HPV(+)OPSCC, acknowledged as a novel subtype in the 4th World Health Organization (WHO) classification,^1^ originates from HPV infection in normal epithelium, eventually undergoing malignant transformation. Notably, HPV(+)OPSCC exhibits dissimilar characteristics compared with HPV‐negative head and neck squamous cell carcinoma [HPV(−)HNSCC], which primarily arises due to tobacco or alcohol‐induced genomic alterations.^2^ A comprehensive genomic investigation conducted by Gillison and colleagues revealed that HPV(+)OPSCC presents a distinctive molecular profile characterised by a lower frequency of TP53 mutation and an elevated occurrence of PIK3CA copy number variations (CNVs).^3^ HPV(+)OPSCC patients are typically younger, non‐smokers and non‐drinkers, experience more favourable overall survival rates and demonstrate heightened sensitivity to radiotherapy.^4^ More recent studies including our own findings have demonstrated a steady annual increase of 5% in the prevalence of HPV(+)OPSCC.^5^ Furthermore, a subset of HPV(+)OPSCC patients still develop recurrence or treatment failure despite standard therapy, underscoring the urgent need for a better understanding of the mechanisms underpinning HPV‐driven carcinogenesis and the development of innovative therapeutic approaches.^5^, ^6^, ^7^, ^8^

The well‐established mechanism of HPV‐driven carcinogenesis involves the action of HPV oncoproteins E6 and E7. These oncoproteins play a crucial role by targeting tumour suppressor proteins p53 and Rb, respectively, resulting in their degradation and leading to genomic instability and the eventual onset of carcinogenesis. Paradoxically, E6/E7 oncoproteins also sensitise tumours to radiotherapy by inducing DNA damage and inhibiting DNA damage repair process.^9^ The instance of standard therapy failure in certain patients suggests the existence of mechanisms independent of E6/E7 oncoproteins. One such mechanism involves the integration of viral breakpoints into the host genome, a pivotal step in the carcinogenesis process. The role of viral integration in tumourigenesis extends beyond HPV and is observed in other viruses such as hepatitis B virus and Epstein–Barr virus.^10^, ^11^ In liver and nasopharyngeal carcinoma, these viruses have demonstrated a propensity to integrate into vulnerable regions of the host genome, leading to genomic instability.^10^, ^11^ In HPV‐associated cervical cancer (CC), emerging studies have revealed the presence of HPV as episomes in the initial stage of infections.^12^, ^13^, ^14^ As the tumour progresses, HPV DNA integrates into the host genome and interacts with it to cooperatively shape the tumour's molecular characteristics.^3^, ^12^, ^13^, ^14^ Whole genome sequencing (WGS) studies revealed that HPV integration can cause genomic alterations, including CNVs and structural variations,^14^ while long‐read sequencing further confirmed the co‐occurrence of viral integration and host genome amplification or rearrangement.^15^, ^16^ Frequently mutated genes in HPV‐related cervical carcinoma (CC) and OPSCC, such as PIK3CA, PTEN, EP300 and FBXW7 suggested a potential role of HPV in shaping host genomic characteristics.^3^, ^17^ However, the distinct gene mutation profiles underscore the varying effects of HPV integration on shaping the molecular characteristics of these two tumour types. Furthermore, the unique viral–host interaction seen in HPV(+)OPSCC, originating from crypt epithelium rather than the surface epithelium of oral mucosa as in HPV(−)HNSCC, and usually characterised by increased lymphocyte infiltration, underscores the importance of investigating HPV integration events in OPSCC.^18^

In addition, recent studies have highlighted that viral integration can lead to the formation of super‐enhancer‐like elements, subsequently inducing changes in both mRNA transcript and protein expression levels of host genes in HPV(+)CC cell lines.^19^ However, the specified roles of HPV integration in regulating host gene expression remain insufficiently investigated warning further exploration to understand the rules and effects of HPV integration in driving tumour progression. Consequently, a thorough investigation into the characteristics of HPV integration in OPSCC can strengthen our understanding of the underlying mechanisms of tumourigenesis in this specific tumour.

Previous studies have primarily focused on the identification of HPV integration sites within HPV(+)OPSCC, employing WGS or RNA sequencing data.^13^, ^14^, ^20^ However, limited sequencing depth and sensitivity restricted a thorough investigation of integration events and the identification of consistent HPV integration patterns. RNA‐based detection methods may mis‐map the corresponding integrant templates.

To overcome these limitations, high‐throughput viral integration detection (HIVID) method which specifically capture HPV DNA sequence was employed in this study. In this study, we conducted a viral integration study using a large cohort of 109 HPV(+)OPSCC tumours with HIVID to identify HPV integration patterns and map the integration sites within the host genome. Furthermore, we performed targeted DNA sequencing on 605 cancer‐related genes, whole exome sequencing (WES) and RNA sequencing to assess the impact of HPV integration on the host genome. Collectively, our study provides insights into clinically relevant HPV integration events, which may facilitate the development of effective treatment strategies for HPV(+)OPSCC.

## 2 MATERIALS AND METHODS

### 2.1 Sample and relative clinicopathological information collections

Over the period of 2008−2020, 109 HPV(+)OPSCC tumours were collected along with relevant clinicopathological information (age, gender, primary tumour sites, tobacco and alcohol history and lymph node metastasis status) from the Shanghai 9th People's Hospital, Shanghai Jiaotong University. All samples used for HPV integration analysis were formalin‐fixed paraffin‐embedded (FFPE) tissues. Among the 109 samples, 38 FFPE tissues were subjected to targeted DNA sequencing which targets exon regions of a panel of 605 cancer‐related genes, while another 29 samples with available liquid nitrogen frozen tissues were used for whole exome sequencing (WES) analysis. In these 29 samples which received WES, RNA sequencing was performed on 26 liquid nitrogen tissues with sufficient RNA quantify and quality. (The detection methods each sample received were documented in the Table S1, and the 605 cancer‐related genes were listed in the Table S2.)

### 2.2 HPV breakpoints detection and annotation

The transcriptionally active HPV status of all samples was determined using the gold standard HPV RNA in situ hybridisation (ISH) testing by the RNA Scope HPV Kit (Advanced Cell Diagnostics, Inc., Hayward, CA), to detect HPV E6/E7 mRNA as previously described.^5^ HIVID was performed as reported.^21^ Prepared DNA libraries were hybridised with the HPV probes, which designed by MyGenostics (MyGenostics Inc., Beijing, China), and bound to magnetic beads before being subjected to high‐throughput sequencing on an llumina HiSeq 3000 sequencer (Illumina Inc., San Diego, CA). Reads were deduplicated by using Bammarkduplicates2, and low‐quality and short reads less than 40 bp and reads that aligned perfectly to the either human or HPV genome were excluded. Clean reads were then mapped to the HPV genome to choose optimal HPV strains. Clean reads were then remapped to human and HPV reference genomes, and the integrated HPV breakpoints were identified at the joint position of human and HPV sequences by using SV detect and CREST analysis, and integration breakpoints whose supporting reads≥2 were selected. CREST analysis results were considered high‐confidence, and the consensus sequence close to the integration site was compiled. After supplementing the SV detect analysis results without integration sites detected by CREST with low‐confidence results, we used ANNOVAR to annotate the high‐confidence results and select high‐confidence results, which were used for subsequent analysis.

### 2.3 HPV breakpoints validation by PCR and Sanger sequencing

A total of 90 viral integration sequences were chosen to perform PCR and Sanger sequencing. Sequences derived from human genome and HPV genome at HPV‐host junction were designed as PCR primers based on the assembled paired‐end fragments. Briefly speaking, a total of 25 μL volume containing 1 μL primers, 1 μL DNA, 1 μL dNTP (mix), 2.5 μL Taq Buffer(with MgCl_2_), 0.2 μL Taq polymerase and ddH_2_O was prepared. For PCR reaction, the following reactions were performed: for 5 min, heat to 95°C, followed by 94°C for 30 s; Polymerisation at 72°C for 30 s after 63°C for 30 s. A total of 10 cycles were performed. Heat was applied to 95°C for 30 s, annealing was applied at 58°C for 30 s and 72°C for 30 s was followed. After 30 cycles, keep at 72°C for 10 min. The PCR amplification products were then applied for Sanger sequencing and following analysis with Sequence Analysis.

### 2.4 DNA sequencing and analysis

For the 38 FFPE samples that were subjected to targeted sequencing, the GenCap capture kits (MyGenostics Inc., Beijing, China) were used to capture the amplified DNA, while the DNA probes were used to target the exon regions of 605 cancer‐related genes. Briefly speaking, the Illumina HiSeq X 10 sequencer was used for enrichment libraries sequencing and after alignment to the human reference genome (hg19), Picard (v2.18.7) was used for refinement. For subsequent analysis, Mutect2 (v1.1.6) from GATK and CNV kit (v0.9.3) were utilised to detect somatic mutations and copy number variations (CNV). WES was performed on 29 fresh frozen samples with normal controls using the Agilent SureSelect V6 kit to capture exomes. Novaseq S4 PE150 was used to sequence germline tissues at a depth of 100 and tumour tissues at a depth of 200. The trimmed and filtered reads were aligned to the UCSC human reference genome (hg19) using BWA (v0.7.15), and the alignments were refined using the Picard (v2.18.7) tool for subsequent data analysis. WES data were aligned to HPV breakpoints sequences detected by HIVID to define consistent breakpoints. The Sention with TNhaplotyper algorithm and CNV kit (v0.9.3) were utilised to detect somatic mutations and CNVs in this study. With CNV kit (v0.9.3), CNVs were detected as described in our previous study.^18^ Briefly speaking, sequences reads were mapped to 5 kb bins, and the genomic copy number was estimated. In this study, we defined genes with Log_2_(copy number) −1 > 0.4 as gains, and genes with Log_2_(copy number) −1 < −0.4 as losses according to the CNV kit. After determining the location of CNVs, we mapped HPV breakpoints on the human genome. The location of integration sites and its nearest CNVs were determined as middle sites of their region and then the distance between them was calculated.

Mutations that were identified as PASS status with TNhaplotyper, located within the targeted region, and absent in dbsnp and cosmic, were used for tumour mutation burden (TMB) calculation in each sample. Using mutation counts divided by 35.08 MB target region size, mutations per targeted megabase were calculated. By using Sention with the TNhaplotyper algorithm, we detected all single nucleotide variants (SNVs), followed by annotation using the Picard (v2.18.7). We extracted the 3 nt‐sequence context of identified SNVs (a total of 96 genomic 3‐nt sequences). SomaticSignature was used to deconvoluted all 3‐nt sequences into 30 mutation signatures, which were annotated on COSMIC and represented for different mutation pattern arising from distinct aetiologies Correlations of HPV integration numbers and mutation signatures were tested by Pearson's correlation.

### 2.5 RNA sequencing and analysis

For RNA sequencing (RNA‐seq), 26 fresh frozen tumours were analysed. Truseq stranded mRNA was utilised to prepare RNA libraries which were then sequenced on Novaseq S4 PE150. The reads were trimmed with Skewer (v0.2.2) to remove adaptor containment sequences and low‐quality base. STAR (2.4.2a) was used to align trimmed and filtered reads to the reference transcriptome (hg19). RNA‐sequencing data were aligned to integrated HPV sequence detected by HIVID to define consistent breakpoints. Quantification for gene expression level was performed using RSEM (v1.2.29). The edge R package was used to analyse normalised raw count data. We then mapped clean reads to human genome 19 and quantified the gene expression level by calculating FKPM values. Gene expression values were transformed as log_2_ (1þx) for downstream analysis. For subsequent normalisation, Log_2_(FKPM) values were calculated as representation of gene expression level. For each gene across 26 samples, the mean (*M*) and standard deviation (SD) were calculated based on Log_2_ (FKPM) value, and *Z*‐score = [(*X* − *M*)/SD], where *X* = Log_2_(FKPM) of this gene.

### 2.6 HPV integration events analysis

We investigated the effects of HPV integration events on the host genome and gene expression by analysing the combined data of HPV integration, DNA sequencing, and RNA‐seq of matched samples. In this study, integration sites were defined as the loci where the viral sequence inserted at the host genome. Integration numbers were defined as the exact number of viral breakpoints that integrate into the host genome, and integration events were identified as the detection of chimeric reads that contains segments of both HPV DNA and human genome.

Genomic compartments were defined based on genomic regions annotated explicitly in GENECODE (gencodegenes.org). Integration sites in different compartments were determined by overlapping with the relevant regions.

Hot spot genes were defined as those exhibiting a significantly higher frequency of viral breakpoints insertion than expected by chance. To determine these hot spot genes, we employed an in silico approach to generate random integration sites. The anticipated number of insertions within a given gene range (within ± 500 kb) was calculated based on the proportion of the gene's span within the entire genome. The actual number of integration sites within each gene was quantified using the HIVID method, and this was designated as the observed count. Subsequently, chi‐square goodness‐of‐fit was employed to test the observed counts with a simulated distribution of integrations per gene. The specific criteria for defining hot spot genes was established based on this analysis.

The distance between integration sites and CNV‐characterised segments was calculated and termed the ‘observed distance’. By performing a permutation test to randomly redistribute all integration sites uniformly across the genome, the calculated distances between the simulated integration sites and the nearest CNVs were determined and defined as ‘expected distance’. For integration sites within CNV regions, their distance to CNVs was recorded as 0. Then, we divided the distances into segments of 10 kb and calculated the frequency distribution of integration sites within each segment. Differences between the observed and expected distances were statistically compared by using the Mann–Whitney test.

Gene expression values were calculated as Log_2_(FKPM) for 26 samples after RNA sequencing. Expression values of samples with viral breakpoints insertion (within ± 500 kb) were compared with samples without HPV integration. The Mann–Whitney test was used to assess statistical significance of gene expression levels between samples with and without integration, and of genes with significant differences (*p* < .05) between the two groups, genes with absolute *Z*‐score value of expression level ≥2 were identified as ‘outlier genes’, indicative of their co‐occurrence with HPV integration. We also identified genes exhibiting both outlier expression and CNVs with HPV integration by comparing copy number data of genes across all tumours, focusing on those genes inserted by HPV breakpoints and simultaneously exhibiting outlier expression levels.

### 2.7 Functional enrichment testing

Enriched Gene Ontology (GO) terms and Kyoto Encyclopedia of Genes and Genomes (KEGG) pathways were explored using RNA‐Enrich (http://lrpath.ncibi.org/), to investigate significantly and highly integrated gene sets (i.e., genes with higher viral integration numbers than expected randomly or by chance), and takes into account any relationship between gene read count and significance level. The directional RNA Enrich test was also performed to identify significantly highly integrated gene sets. Custom code was employed to decrease redundancy (for example, GO terms without significance and related closely) for presenting the top enriched terms.

### 2.8 Comparison of copy numbers and expression values of genes with and without HPV integration

Quantile–quantile (*Q*–*Q*) plots were used to analyse distribution of gene copy numbers and expression values of genes with and without HPV integration, respectively. Genes with HPV integration within ± 500 kb were identified as genes with HPV integration, and the *Z*‐score of RNA expression of each gene was calculated and normalised. The quantile of Log_2_(Copy number)−1 and normalised *Z*‐score values of genes without HPV integration were utilised as horizontal axes, while the corresponding quantile values of the same genes with integration were used as vertical axes, and then, a scatter plot (marked as red dot in this study) was drawn. Parametric curve was drawn on the hypothesis that genes with and without viral insertion have similar copy number and expression values. The R package for drawing *Q*–*Q* plot is available on https://github.com/stephenturner/qqman. The Kolmogorov–Smirnov test was used to test whether the distribution of gene copy number and RNA expression level of genes with viral integration fits the distribution of genetic copy number and RNA expression level of genes without viral integration.^14^

## 3 RESULTS

### 3.1 Landscape and characteristics of HPV integration in the human genome

**FIGURE 1:**
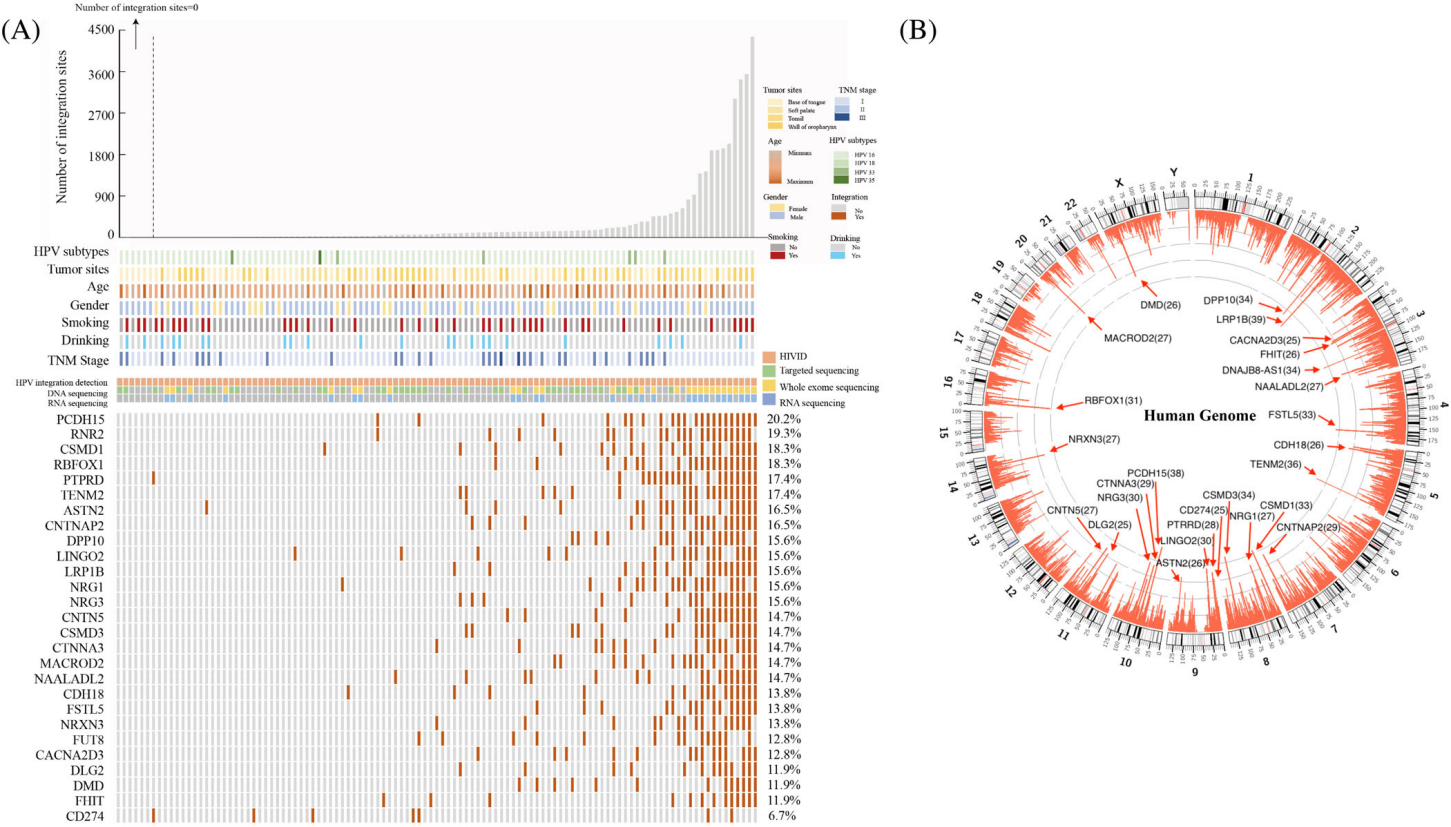
Landscape of HPV integration on the host genome. (A) Clinical annotation of HPV integration across 109 samples. Each vertical track represents an individual sample. The upper histogram depicts the count of HPV integration sites in each sample, and the middle heatmap presents clinicopathological patient information and detection methods used for each tumour. The bottom heatmap shows distribution of recurrent integration sites across different samples, with numbers indicating integration frequencies in genes being integrated across 109 samples. (B) Distribution of HPV integration sites in the human genome (hg 19) among 103 samples with viral integration. Each bar of outermost circle denotes integration position into the human genome. Red bars of the inner circle indicate integration frequency at specific loci, with higher frequency sites highlighted for reference, and the histogram axis units represents number of integration.

In this study, we conducted HIVID analysis (Table S1) on 109 HPV(+)OPSCC samples (Confirmed by HPV RNA in situ hybridisation, Figure S1A, and the workflow diagram of HIVID was illustrated in Figure S1B.) The clinical data related to the samples are shown in Table 1 and Figure 1A. Among the 109 cases, HPV viral typing showed that 93.6% (102 out of 109) were HPV16, 4.6% (five out of 109) were HPV18, 0.9% (one out of 109) were HPV33 and 0.9% (one out of 109) of the cases were HPV35. We detected total of 36 751 HPV breakpoints in 103 of 109 HPV(+)OPSCC samples (Table S3), and 87.8% (79 out of 90) randomly chosen integration sites were validated successfully using PCR and Sanger sequencing (Table S4 and Figure S1C), while DNA and RNA sequencing also confirmed HPV breakpoints in the human genome (Figures S1D–S1G). To explore the association between viral integration and clinicopathological characteristics, we examined the integration numbers and patients’ parameters. Notably, tumours from the tonsil or oropharyngeal wall regions with lymphoid follicles and high PD‐L1 expression (58.5% of tumour arising at tonsil or oropharyngeal wall showed high PD‐L1 expression vs. 13.6% of tumour arising at base of the tongue or soft palate showed high PD‐L1 expression, *p* = .002) exhibited higher integration numbers compared with those from the base of tongue or soft palate (mean 434 vs. 194 per sample, *p* = .024). This suggests that the potential role of tumour microenvironment with elevated levels of immune evasion status might contribute to viral integration. Integration sites distribution was also explored across independent samples, revealing common integration spots like PCDH15 (22 of 109 samples, 20.2%), RNR2 (21 out of 109, 19.3%), CSMD1 (20 out of 109, 18.3%), LRP1B (17 out of 109, 15.6%), FUT8 (14 out of 109, 12.8%), CD274 (seven out of 109, 6.7%) and so on. These recurring spots indicated preferred loci for viral fragment involvement. We also mapped HPV integration sites in the human genome (Figure 1B), which revealed a broad integration pattern with frequent sites. Interestingly, more than 20 integration events were identified in 62 genes across all samples, such as LRP1B (39 times), PCDH15 (38 times), TENM2 (36 times) and so on. OPSCC demonstrated significantly more integration events than CC (mean integration number: 337 vs. 27 per sample, *p* < .0001).^12^ In addition, repeated integration events were observed in the same samples, such as HPV‐CD274 (encoding PD‐L1 protein). Although the integration of CD274 occurred in only seven samples, it appeared four times in sample S011, twelve times in sample S033, five times in sample S090, and once each in samples S009, S031, S100 and S112. 85.7% (6/7) of these samples exhibited high PD‐L1 expression (combined positive score > 20), indicating the impact of HPV integration on gene expression (Figure S2).

**TABLE 1:** Demographic and clinicopathologic characteristics of HPV(+)OPSCC patients and their association with HPV integration.

Variable	Number of patients	Mean number of integrations	*p* Value (Mann–Whitney *U*‐test)
Age			
≤57	59	419.4	
>57	50	240.2	*p* = .836
Smoking status			
No	70	248.5	*p* = .754
Yes	39	496.3	
Drinking status			
No	86	357.6	*p* = .185
Yes	23	260.8	
Tumour origin site			
Tonsil or oropharyngeal wall	65	433.8	*p* = .024
Non‐tonsil or oropharyngeal wall	44	194.4	
LN metastasis			
No	19	76.7	*p* = .203
Yes	90	392.2	

### 3.2 HPV integration shows predisposition in human genome regions

**FIGURE 2:**
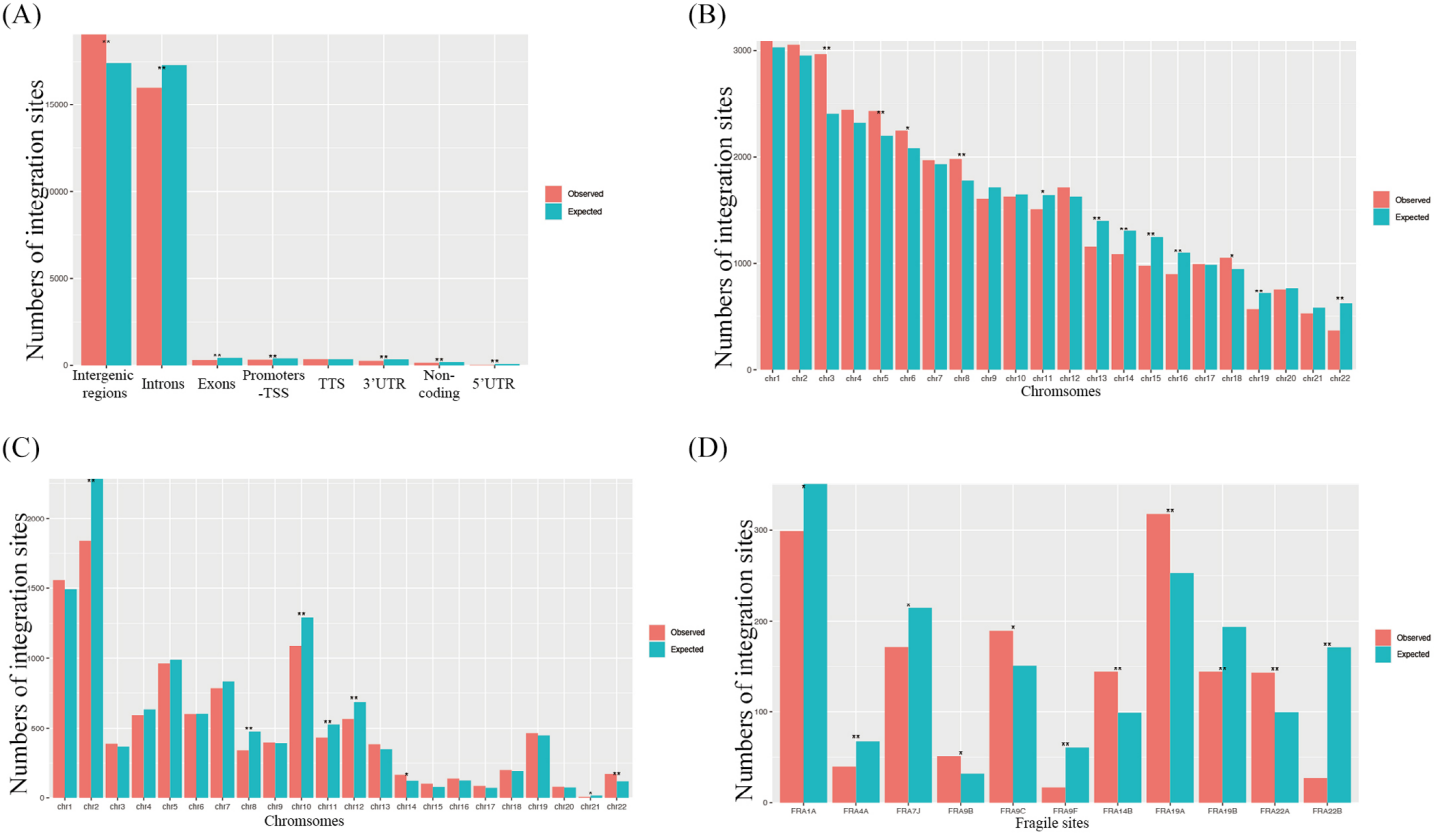
Predisposition of HPV Integration on the Host genome. (A) Assessment of HPV integration sites in distinct regions of the human genome. Comparison between expected (green bar, assuming uniform, random distribution) and the observed (actual numbers, red bar) numbers of HPV integrations. TSS, transcription start site; TTS, transcription termination region; UTR, untranslated regions. (B) Analysis of HPV integration sites across human chromosomes. Observed integration site counts (red bars) are compared with expected counts (blue bars), assuming uniform, random distribution. (C) Evaluation of viral breakpoints within fragile sites on human genome. Observed integration site counts (red bars) are compared with expected counts (blue bars, assuming uniform, random distribution). (D) Assessment of HPV integration sites within specific fragile sites. Comparison the expected (green bar, uniform, random distribution,) and observed (red bar) HPV integration counts. *p* Values were calculated using the Chi‐square test. ‘*’ indicates *p* < .05. ‘**’ indicated *p* < .01.

To explore the distribution pattern of HPV‐DNA across the human genome, we examined integration predispositions. We found that integration events often occurred in intergenic regions significantly within distinct genome areas (Figure 2A). Furthermore, integration event were notably concentrated on chromosomes 3, 5, 6, 8 and 18 (*p* < .05, Figure 2B), suggesting a tendency for integration at the chromosome level, which led us to hypothesise that there is a selection advantage during HPV integration, leading to recurrent integration at similar sites in different samples.

We also investigated whether viral integration correlated with fragile regions of the human genome, specifically fragile sites (FSs). FSs are known for increased genomic instability and are categorised as common fragile sites and rare fragile sites. Interestingly, HPV integration was detected in 117 FSs, with chromosomes 14 and 22 showing significant integration (Figure 2C). Certain FSs, such as FRA19A (observed integration sites vs. expected integration sites = 318 vs. 253, *p* = .006), FRA9C (observed integration sites vs. expected integration sites = 189 vs. 151 *p* = .041), FRA14B (observed integration sites vs. expected integration sites = 144 vs. 99, *p* = .004) and FRA22A (observed integration sites vs. expected integration sites = 143 vs. 99, *p* = .005), exhibited noteworthy enrichment of integration (Figure 2D). The predisposition of HPV integration at some FSs suggests that viral breakpoints are more likely to insert at certain unstable loci in the human genome, highlighting its relevance in HPV(+)OPSCC. This provides valuable insight for investigating viral integration mechanisms.

### 3.3 Genes with integration predisposition are enriched in cancer‐related genes and involved in specific functional pathways

**FIGURE 3:**
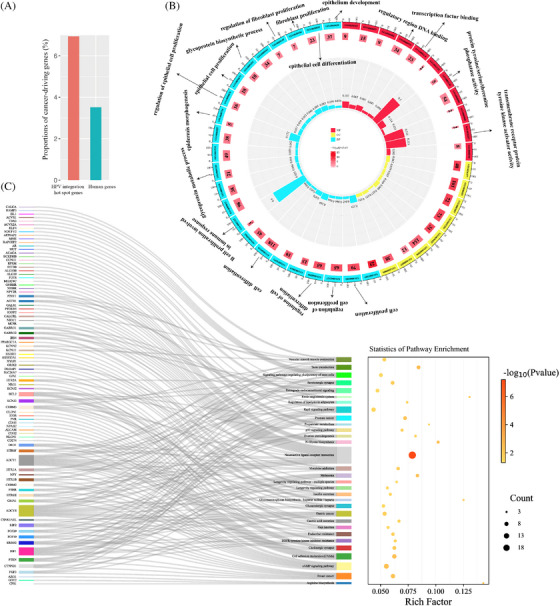
Functional implications of integration hot spot genes. (A) Comparison of cancer‐driving genes within the significant integration hot spot genes (red) and in the human genome (green). (B) Gene Ontology (GO) analysis for the integration hot spot genes with significant high frequency. Numbers within middle circle boxes represent counts of enriched genes for each term, with arcs at the outside indicating measurement. Box colours signify different p‐values, while histogram colours correspond to rich factor values as labelled. (C) Analysis of Kyoto Encyclopedia of Genes and Genomes (KEGG) pathways for integration hot spot genes with significant high frequency.

HPV integration sites exhibited scattered distribution across the human genome, with some HPV breakpoints being dispersed randomly on human chromosomes, while others were notably concentrated within specific genes. To explore this phenomenon, we compared simulated integration numbers of genes undergoing random integration and the observed integration numbers, followed by statistical testing. We identified a total of 611 coding genes displaying significantly enriched HPV breakpoints (Chi‐square goodness‐of‐fit, *p* < .05), and these genes were designated as integration hot spot genes in this study. Among the protein coding genes within these integration hot spot genes, 6.9% (30 out of 432) were represented by cancer‐driving genes from the COSMIC Cancer Gene Census while only 3.5% (712 out of 20 242) of protein coding genes within the entire human genome encompassed cancer‐driving genes (Figure 3A, Chi‐square test, *p* = 5.2e−4).

To better understand integration hot spot genes’ biological and functional significance, we conducted a GO analysis and KEGG pathways enrichment analysis. Our GO analysis uncovered the enrichment of genes associated with many biological functions, particularly those related to epithelial processes (Figure 3B), including epithelial cell differentiation (*p* = .005), epithelial cell proliferation (*p* = .001) and epithelium development (*p* = .008). Moreover, we observed significant enrichment in immune‐related biological processes, such as B cell proliferation involved in immune response (*p* = .0001), and glycoprotein biosynthetic process (*p* = .001) (Figure 3B). KEGG pathway enrichment analysis further emphasised the significance of integration hot spot genes, particularly their enrichment in N‐glycan biosynthetic processes such as FUT8 and STT3B. These genes were integrated for 24 and 11 times, respectively, and are known for pivotal vital roles in tumour immune evasion (Figure 3C). Overall, these findings provide additional support for the preferential integration of HPV within cancer‐related genes, potential influencing epithelial and immune‐related biological processes and implying that the integration of HPV in functional gene sites may contribute to driving carcinogenesis.

### 3.4 Distributions of viral breakpoints in HPV genome

**FIGURE 4:**
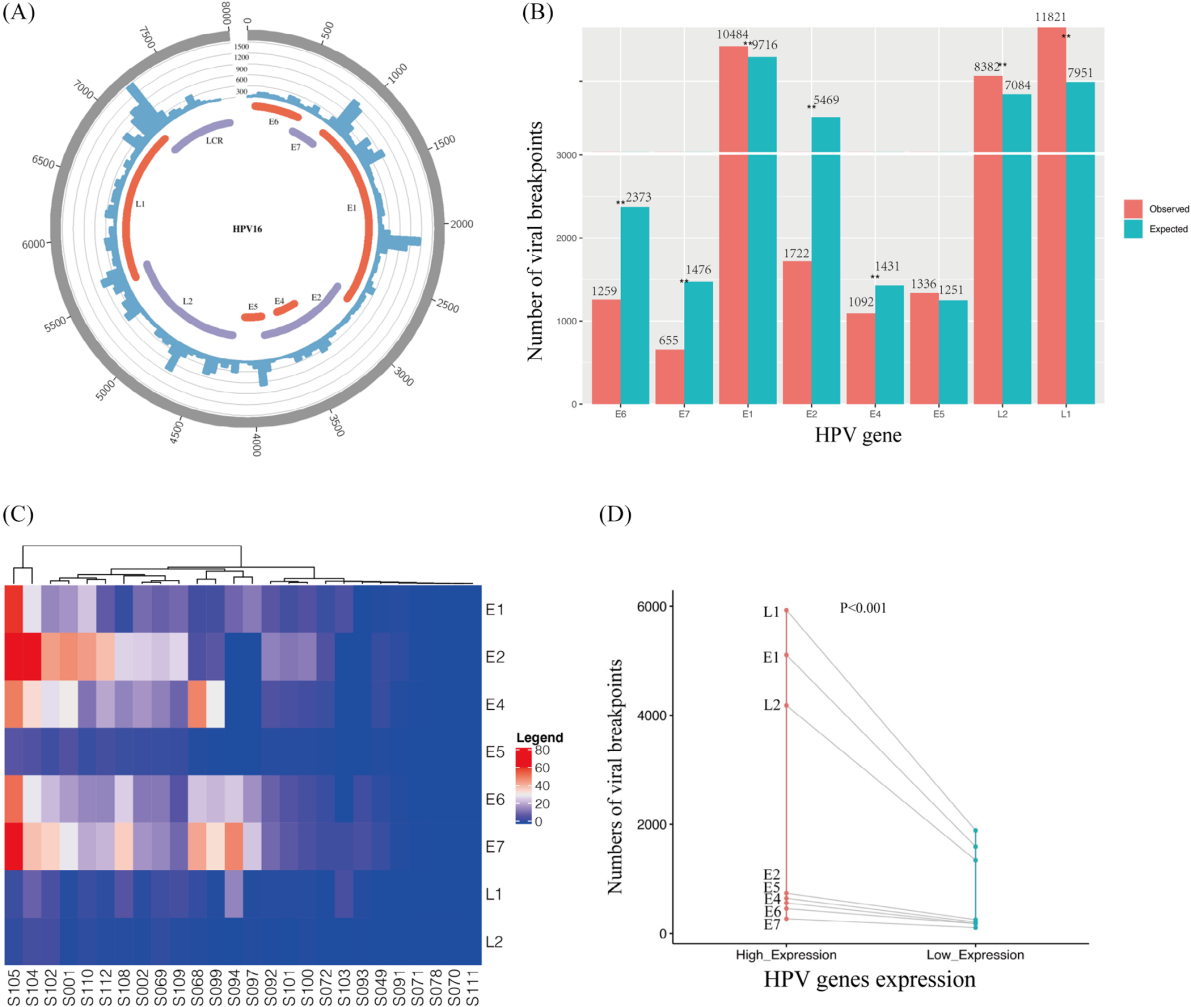
Distribution of HPV breakpoints on the HPV genome. (A) Distribution of viral breakpoints within the HPV16 genome across 93 samples. Histograms depicts the frequency of breakpoints in the samples. Histogram axis units represent breakpoints numbers. (B) Comparison of observed (red) and expected (green) numbers of breakpoints in the HPV genome. *p* Values were determined through Chi‐square test. ‘*’ indicates *p* < .05. ‘**’ indicates *p* < .01. (C) Heatmap demonstrating the expression levels of HPV genes in 26 samples subjected to RNA sequencing. (D) Comparison of breakpoint counts for each HPV gene between samples with high and low viral gene expression.

To gain further insights into the integration pattern within the HPV genome, we mapped viral breakpoints on the HPV genome. We then calculated the frequency of integration within each viral genome region from a total of 103 samples with HPV integration. Among these, 93 HPV16 (AF534061.1, AF125673.1, HQ644236.1, AF536179.1, marked in the Table S1) subtype‐positive samples sharing similar HPV genome were collectively mapped in Figure 4A, while the remaining three HPV16 (NC001526.4), five HPV18 (AY262282.1; GQ180784.1; GQ180789.1), one HPV33 (HQ537691.1) and one HPV35 (HQ537713.1) subtype‐positive samples were individually mapped in Figure S3. Frequency calculations of breakpoints in each viral region revealed that the HPV genome could experience breaks across its entirety, however, it exhibited a higher likelihood of breaking in areas E1, L1 and L2 (*p* < .001) (Figure 4B). Furthermore, we observed a significant tendency for regions E6, E7, E2 and E4 to remain intact (*p* < .001), consistent with the preservation of their oncogenic roles during HPV(+)OPSCC carcinogenesis and viral cycling. Thus, we hypothesised that these genes might be conserved to promote malignant transformation in host cells. Comparatively, the L1 and L2 regions were more prone to break and integrate into host cells (*p* < .001), potentially acting as cis‐activators of nearby host genes and regulating their expression. Among the 26 samples that underwent RNA sequencing, we divided them into high and low viral gene expression groups based on their level of viral gene transcript expression (Figure 4C). Samples within the high viral gene expression group exhibited a significantly higher number of viral breakpoints than those in the lower viral gene expression group (Figure 4D, *p* < .001 and Table S5), suggesting that viral integrations into the host genome promote viral expression and facilitate HPV‐driven carcinogenesis.

### 3.5 Molecular characteristic of HPV(+)OPSCC and association of HPV integration and CNVs

**FIGURE 5:**
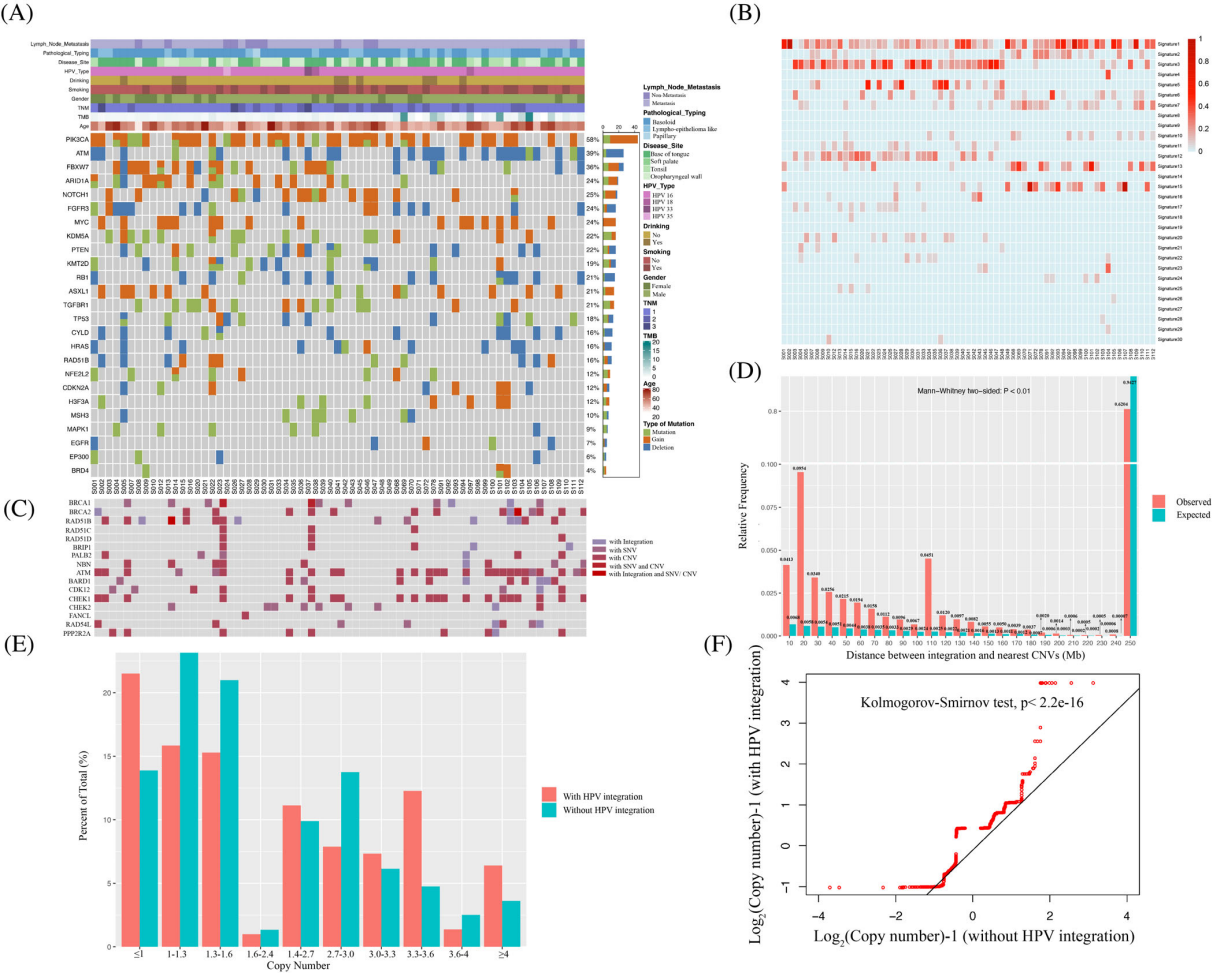
Associations between HPV integration and host genomic alterations. (A) Heatmap depicting variants in mutated genes across 67 samples. Orange indicates gene copy number gain, blue signifies copy number loss and green denotes somatic variants including missense single nucleotide variants (SNVs), nonsense SNVs, splice variants, in‐frame insertions, deletions, frame‐shift deletions and frame‐shift insertions. The accompanying table on the right displays the percentages of samples with SNVs and CNVs (combined). Individual genes are ranked based on these combined frequencies from top to bottom. (B) Heat map illustrating the fractions of mutation signatures across 67 samples. (C) Distribution of HPV integration and variants in homologous recombination repair (HRR)‐related genes. (D) Comparative histograms of observed and expected genomic distances between HPV integration sites and the nearest CNVs (Mann–Whitney test). (E) Distinct frequency distributions (*y*‐axis) of copy numbers (*x*‐axis) of genomic segments with HPV integration (red) versus those without (green) HPV integration across 67 samples. (F) Quantile‐quantile (*Q*–*Q*) plot confirming differences in copy numbers of genomic segments with viral integration (*y*‐axis) compared with those without (*x*‐axis) HPV integration, significantly deviating from the line of identity (*p* < 2.2e−16, Kolmogorov–Smirnov test).

We analysed DNA sequencing data from 38 FFPE HPV(+)OPSCC tumour samples and 29 fresh frozen HPV(+)OPSCC tumour‐normal pairs with relevant clinicopathological information (Figure 5A). The landscape of HPV(+)OPSCC genome features showed that CNVs accounted for most tumour genomic alterations. PIK3CA, which is associated with APOBEC‐mediated mutagenesis, was the most frequently altered gene in HPV(+)OPSCC, with a copy number gains rate of 52.2% and mutation rate of 13.4%. A total of 8112 somatic variants were detected, including 4863 missense SNVs, 437 nonsense SNVs, 138 splice variants, 73 in‐frame insertions, 138 deletions, 208 frame‐shift deletions and 99 frame‐shift insertions. Analysis of SNVs across all tumours demonstrated a relatively low TMB, with a mean of 2.21 mutations per megabase.

The distribution of 30 mutation signatures was then compared (Figure 5B) with the rates of SNVs being calculated in 96 three‐nucleotide genomic sequence contexts (Figure S4A). Notably, mutation signatures 2 and 13 (associated with the APOBEC family, with potential to inhibit HPV infection activity) were enriched in 67 samples. The strong correlations between TMB and mutation signature 2 (*R* = 0.369, *p* = .002, Figure S4B) and 13 (*R* = 0.577, *p* < .0001, Figure S4C) suggested that APOBEC editing played a potential role in HPV(+)OPSCC mutation. It should be noted that the number of HPV integration was also associated significantly with mutation signature 13 (*R* = 0.274, *p* = .025, Figure S4D). Additionally, we observed a negative relationship between HPV integration numbers and signature 3 (*R* = −0.342, *p* = .005, Figure S4E), which is triggered by homologous recombination deficiency (HRD).^22^ Thus, we mapped HPV integration events and homologous recombination repair (HRR)‐related genes’ alterations, which are the main causes of HRD. As shown in Figure 5C, there was a high frequency of alteration in HRR‐related genes. However, HPV integration was found to be seldom integrated into these alterations, as co‐occurrence was only observed in RAD51B in sample S013 and BRCA2 in sample S103.

To evaluate the potential relevance between HPV integration and the distribution of CNVs, we compared the true distance between integration and CNVs with that which might have occurred by chance based on DNA sequencing data of 67 samples. Strikingly, we found that HPV integration occurred significantly closer to CNV than expected by chance (*p* = .0011) (Figure 5D), supporting the idea that HPV integration is an inducement of host genomic CNVs. Since HPV breakpoints were found to be closely inserted into genomic regions with CNVs, we further investigated the relationship between HPV integration and CNVs. The distribution frequency of CNV in the human genome was found to be significantly different in host genome segments with viral fragments compared with those without HPV integration. CNVs were especially abundant in segments harbouring viral integration. As depicted in Figure 5E, 21.51% of genomic segments with viral breakpoints exhibited copy number ≤1, while 13.88% of genomic segments lacking viral breakpoints exhibited copy number ≤1. Additionally, 6.40% of genomic segments with viral breakpoints exhibited copy number ≥4, whereas 3.61% of genomic segments without viral breakpoints had copy number ≥4. This observation highlights significant differences in CNV distribution between host genes with and without viral insertion (Chi‐square test, *p* < 2.2e−16). To further examine the relationship between CNV and HPV integration, *Q*–*Q*) plots were drawn to compare the distribution of CNVs in genes affected by HPV (genes that harbour HPV breakpoints within ± 500 kb) by combining DNA sequencing and integration data of all 67 samples that received DNA sequencing. The results revealed that genes with HPV integration displayed a significantly different frequency of CNVs than those without HPV integration (*p* < 2.2e−16, Figure 5F), suggesting that HPV integration plays a role in the host genomic CNV.

### 3.6 Identification of integration sites co‐occurred with CNVs

**FIGURE 6:**
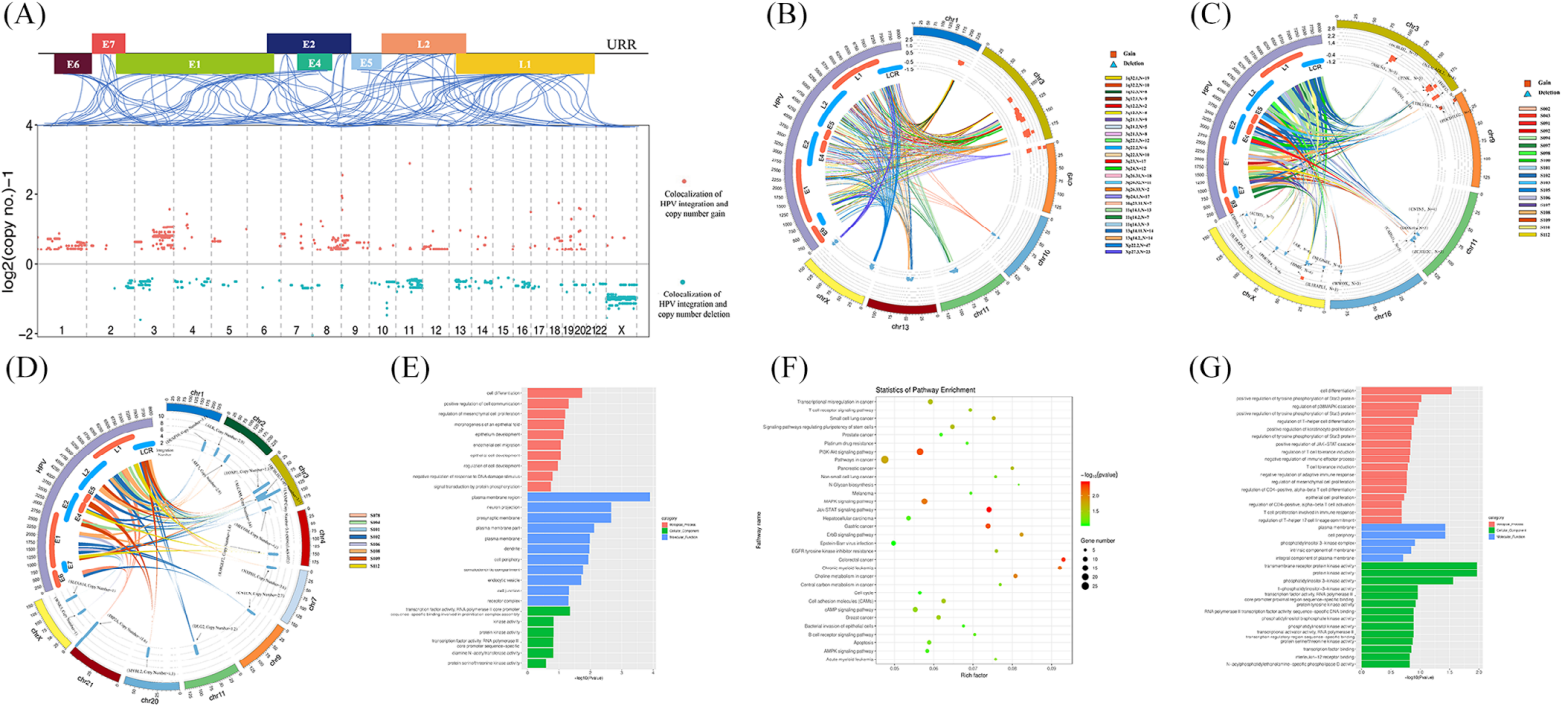
Characterisation of HPV integration sites co‐occurred with host genomic CNVs. (A) Distribution of integrated genes co‐located with nearby CNVs in human genomes. The blue linking lines indicate the location of integration breakpoints on the human chromosome and the HPV genome. The red dot represents copy number gained genes with viral integration nearby, while blue dots indicated copy number deleted genes with viral integration nearby. (B) Circos plot showing the connection between the HPV genome and human chromosome cytobands exhibiting a high frequency of viral integration and CNVs. In the inner circle, histogram units denotes log_2_(copy number)−1. The red squares indicate positions of copy number gained genes with viral integration, and the blue triangles indicate positions of deleted genes with viral integration. (C) Circos plot showing connections between the HPV genome and specific human genes with a high frequency of viral integration and CNVs across all samples. In the inner circle, histogram units represent log_2_(copy number)−1. The red squares indicate positions of copy number gained genes with viral integration. The blue triangles indicate positions of deleted genes with viral integration. “N” indicates the number of samples with co‐occurrence of CNVs and HPV integration in this gene. (D) Circos plot illustrating the connections between the HPV genome and specific human genes exhibiting a high frequency of viral integration and CNVs in one specific sample. In the inner circle, histogram units represent the number of integration sites in this gene of the sample. (E) Gene Ontology (GO) analysis for genes exhibiting co‐occurrence of viral integration and copy number deletion. (F) Kyoto Encyclopedia of Genes and Genomes (KEGG) pathway analysis for genes exhibiting co‐occurrence of viral integration and copy number gain. (G) GO analysis for the genes exhibiting co‐occurrence of viral integration and copy number gain.

We further analysed recurrent copy number gain or deletion regions in 29 samples which received WES in Figure S5. The results showed that many integration sites were located near CNVs, with gain at 9p24.1, 18q21.33, 3q29 and 14q11.2 co‐occurring with viral integration, and deletions at 1q21.3, 2q36.3, 10q23.31 and 9p24.3 were also found to co‐occur with viral integration. To gain further insights into the effects of HPV integration on the host genome, we combined HIVID and DNA sequencing of 67 samples which received DNA sequencing. The results indicated that a cluster of genomic loci with HPV integration exhibited CNVs in independent samples. Specifically, we identified 1893 integration sites located at genomic sites with CNVs in 32 samples, and identified 1292 genes in 410 cytobands (Figure 6A), in which 13.2% showed an integration of more than 10 times, some of which were demonstrated in Figure 6B. These genes with co‐occurrence of viral integration and CNVs in HPV(+)OPSCC exhibited distinct copy numbers compared with the same genes in HPV(−)HNSCC samples derived from TCGA (Figures S6A and B, Kolmogorov–Smirnov test, *p* < 2.2e−16).

Identification of HPV integration hot spot genes demonstrated their notable enrichment. Building upon this discovery, we aimed to explore the potential correlation between these hot spot genes and co‐occurring CNVs. Our analysis revealed specific genomic loci that recurrently exhibited integration and CNV co‐occurrence, observed in multiple independent samples. A subset of these loci is visualised in Figure 6C, implying that HPV integration could potentially provide a selective advantage within particular hotspots, leading to genomic variability and consequent tumourigenesis. Specifically, NAALADL2, located at Chromosome 3q26.31 and previously identified as an integration hot spot genes in Figure 1A, was found to co‐occur with gene gain in four samples (S094, S100, S102, S108). Notably, the viral integration site NLGN1, which is adjacent to NAALADL2, also exhibited co‐occurrence with gene gain in four independent samples (S094, S097, S100, S102). Both NAALADL2 and NLGN1 reside within 3q26.31, a FSs prone to frequent alterations.^23^ CNVs at this site may activate its nearby oncogenes such as ATR, BCL6 and PI3K family, leading to aggressive cancer hallmarks in prostate cancer.^24^ As previously mentioned, 3q26.31, which contains genes NAAlADL2 and NLGN1, was identified as an integration hot segment with copy number gain. Based on this finding, we hypothesised that specific host genome cytobands might serve as integration hot spot genes associated with CNVs (Figure 6B), suggesting that HPV integration correlated with host genome instability. We also observed a cluster of HPV breakpoints enriched in specific genes in one sample with CNVs (Figure 6D). For example, HPV segments were found to integrate at DIP2A 12 times in sample S078, followed by a 31.6‐fold amplification of DIP2A. Overexpression of DIP2A has been reported to elevate follistatin‐like 1 (FSLT1) expression in mouse tumour model, inducing immuno‐resistance consequently.^25^ As this tumour showed an atypical clinicopathological characterisation, including high‐differentiated pathological morphology and poor prognosis (of note, the patient relapsed after radiotherapy), and showed low levels of HPV transcript expression (Figure 4C), the carcinogenesis mechanism of this tumour may be contributed by integration events rather than traditional HPV oncoproteins. Thus, we conclude that some functional genes might confer a selective growth advantage for viral integration, further accelerating carcinogenesis.

We hypothesised that genes close to viral integration might be potentially disrupted by integration, leading to their deletion. To investigate this further, we conducted GO analysis and found that these genes play important roles in vital functions, such as cell differentiation, epithelial cell development and negative regulation of response to DNA damage stimulus (Figure 6E), including Androgen receptor (AR), which was observed to be inserted in six independent samples and showed copy number deletion (Figure 6C). Except for AR, some sites were found to be repeatedly integrated in different samples with copy number deletion, some of which are demonstrated in Figure 6C, such as Actin Related Protein T1 (ACTRT1) in chromosome Xq25. Loss of ACTRT1 function was reported to lead to aberrant activation of Hedgehog signalling in basal cell carcinoma.^26^ Another example is Forkhead box protein 1 (FOXP1) on chromosome 3p13, which plays a role in T‐cell quiescence and differentiation.^27^ Its deficiency has been reported to be correlated positively with tumour proliferation in prostate cancer or tumour origin in non‐small cell lung cancer.^28^, ^29^ These results suggest that HPV integration in the host genome may lead to the loss of function of genes that serve as tumour suppressors, thus accelerating tumourigenesis. We also observed the co‐occurrence of HPV integration and copy number gain of this gene, which were found to play important roles in transcriptional mis‐regulation in cancer, N‐Glycan biosynthesis, epithelial cell proliferation, T cell proliferation involved in immune response and so on (Figures 6F and 6G). For example, the integration of PDCD1LG2 in samples S102 and S105 was followed by 11.8 and 3.2 fold amplification of this gene, which encodes an important immune checkpoint molecule (programmed death ligand 2, PD‐L2). This may contribute to immune evasion in these tumours. Overall, these findings suggests that the existence of hot spot genes in different tumours that exhibit a predisposition of viral insertion, and further viral integration of these hot spot genes, followed by CNVs in vital genes, may facilitate carcinogenesis.

Sample S094 and S102 serve as striking illustrations of the carcinogenic potential role of HPV integration. As shown in Figure 4D, samples displaying high viral genes expression exhibited a greater number of integration sites. In these two samples (S094 and S102), which exhibited high E6/E7 expression (Figure 4C), a noticeable increase in integration site counts was evident (3433 and 4363 integration sites, respectively). Moreover, 261 and 535 integration sites were identified as coinciding with CNVs in both sample S094 and S102 (Figures S6C and D), suggesting a potential synergistic effect between HPV integration and HPV oncoproteins E6/E7 during the tumourigenesis process. Furthermore, the poor prognosis of these two patients (S094 relapsed after radiotherapy and S102 died from brain metastasis after 3 months of operation) indicates that viral integration may confer distinct carcinogenesis independent of E6/E7, leading to an unusual poor prognosis of HPV(+)OPSCC.

### 3.7 HPV integration associates with outliner host gene expression

**FIGURE 7:**
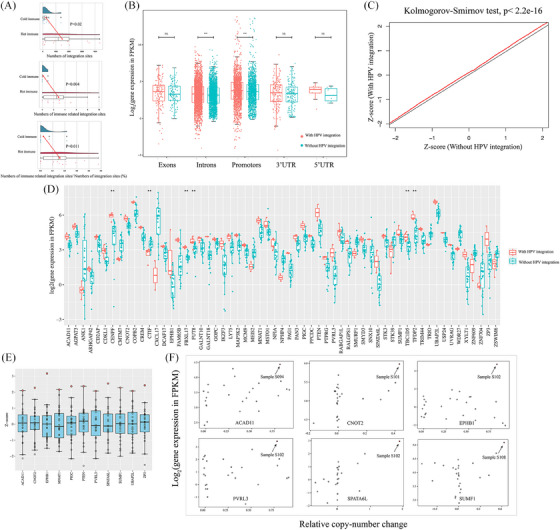
Effects of HPV integration on host gene expression. (A) Comparison of the number of integration sites between cold and hot immune groups (Top). Comparison of the number of immune‐related integration sites between cold and hot immune groups (Middle). Comparison of the fraction of immune‐related integration sites between cold and hot immune group (Bottom). (B) Host gene expression levels categorised by the genomic region with integration occurring (exons, introns, promoters, 3′UTR3 and 5′UTR5). The red boxes for each region represent the gene expression of samples with integration occurring in this region (labelled as ‘integration’); the green boxes demonstrate the average expression of the same genes in all other samples without integration (labelled as ‘without integration’). (C) *Q*–*Q* plot comparing of *Z*‐score distributions of expression levels for genes with HPV integration (±500 kb) (*y*‐axis) versus the expression of the same genes in all other tumours without HPV integration (*x*‐axis) (Kolmogorov–Smirnov test, *p* < 2.2e−16). (D) 59 genes in samples with correspondent virus–host integration (±500 kb within this gene) exhibit different expression levels compared with genes without integration (Mann–Whitney test, *p* < .05, ‘**’ indicated *p* < .01). The red dot indicated genes with integration while the blue dot indicates genes without integration. (E) Of genes with viral integration and exhibiting different expression levels, 11 genes showed outlier expression across all samples (*Z*‐score > 2). (F) Scatter plots comparing copy‐number alterations and gene expression levels in genes with outlier expression and CNVs at integration sites. The black arrow indicates circles represent tumours with HPV integration in these genes.

To investigate the effects of HPV integration on the tumour immune microenvironment, we calculated tumour infiltrating lymphocytes (TILs) score of 26 samples that underwent RNA‐seq, and classified them into two groups: the ‘cold immune’ group exhibiting fewer infiltrating immune cells, and the “hot immune” group highly infiltrated by immune cells (Figures S7A–C). Multiple immunofluorescence analysis shows that tumours in the ‘hot immune’ group have higher numbers of CD68 (mean number: 1142 vs. 656, *p* = .036, Mann–Whitney test) and CD8 (mean number: 2870 vs. 1366, *p* = .031, Mann–Whitney test) positive TILs in stromal regions and CD4 positive TILs (mean number: 2928 vs. 1917, *p* = .027, Mann–Whitney test) in whole slides of area compared with tumours in the ‘cold immune’ group (Figures S7D–L). We hypothesised that the distinction of TILs might be influenced by viral integration and compared the proportion of a panel of immune‐related integration sites in each sample between the two groups. The result indicated that the ‘hot immune’ group had a significantly higher number of integration sites (mean 245 vs. 1486 per sample, *p* = .02, Mann–Whitney test), immune‐related integration number (mean 0.2 vs. 2.9 per sample, *p* = .004, Mann–Whitney test) and a higher percentage of immune‐related gene integration than its counterpart (0.024 vs. 0.160%, *p* = .011, Mann–Whitney test; Figure 7A), thereby supporting the notion that integration affect gene expression and may impact the tumour immune microenvironment. To evaluate the potential impact of HPV integration occurring in different regions on the corresponding host gene, we calculated the expression of all gene transcripts and compared the gene expression level of genes in samples that harbour viral integration with the same gene in all other samples. Notably, the result showed that genes with integration sites near gene introns and promoters were significantly up‐regulated compared with the same gene in other samples (Figure 7B). Therefore, we hypothesised that HPV could be prone to integrate into non‐coding areas and regulate gene expression instead of directly influencing gene function.

As mentioned above, HPV integration may regulate gene expression. To further confirm this, we used *Q*–*Q* plots to compare gene expression levels of those with viral breakpoints nearby (defined as 500 kb distance) versus those without viral breakpoints on 26 samples which received RNA‐seq. Our analysis revealed that genes with viral integration (within ± 500 kb) exhibited significantly different expression levels (Kolmogorov–Smirnov test, *p* < 2.2e−16, Figure 7C). We also observed that some genes with HPV integration had significantly different expression levels compared with the same genes without integration (Mann–Whitney test, *p* < .05). A total of 59 genes were identified, of which 86.4% (51 out of 59) of the genes were associated with malignant tumours (Figure 7D), and among which the expression of 78.0% (46 out of 59) of the genes was elevated. Notably, expression values of these genes impacted by viral integration exhibited distinct expression levels compared with the expression of the same genes in HPV(−)HNSCC derived from TCGA (Kolmogorov–Smirnov test, *p* < 2.2e−16, Figure S8A). GO analysis revealed that these genes play roles in vital functions, such as cellular protein modification process including N‐glycan fucosylation (FUT8), mucin type O‐glycan biosynthesis (GALNT10 and GALNT18), negative regulation of cell cycle process (CENPF, TFDP2, CNOT2, PTEN) and so on, thus confirming HPV breakpoints are associated with cancers and may serve as regulators of oncogene expression and drivers of tumourigenesis (Figure S8B). Among these genes, 11 genes with integration exhibited outlier expression (*Z*‐score of expression value ≥2), further highlighting effects of viral integration on the gene expression (Figure 7E).

As observed above, HPV integration may co‐localise with CNV. We speculate that gene expression variation induced by HPV might result from CNVs promoted by viral integration. As shown in Figure 7F, some integration sites harboured altered copy numbers and dysregulated gene expression. Further, our findings indicated up‐regulated gene expression with viral insertion at the promotors. For instance, in sample S094, we observed viral integration at the promotors of ACAD11, while in sample S102, integration at the promotors of EPHB1, which were all associated with copy number gain. Additionally, in sample S101, we observed integration at the promotors of outlier gene UBAP2L. Based on these observations, we hypothesised that HPV integration might occur at gene promotors, leading to subsequent genomic variations that can affect gene expression. We therefore postulated that genes affected by integration could play an important role in regulating tumour progression. Except for the direct up‐regulation of PD‐L1 that co‐occurred with HPV‐CD274 integration, as observed above, the up‐regulation of FUT8 caused by viral integration was found to have a positive correlation with PD‐L1 and catalysed its glycosylation, facilitating tumour immune evasion (Figure S9). Thus, viral integration‐induced genomic variation may be a probable mechanism for cancer progression, and targeting them could be a promising treatment strategy.

**FIGURE 8:**
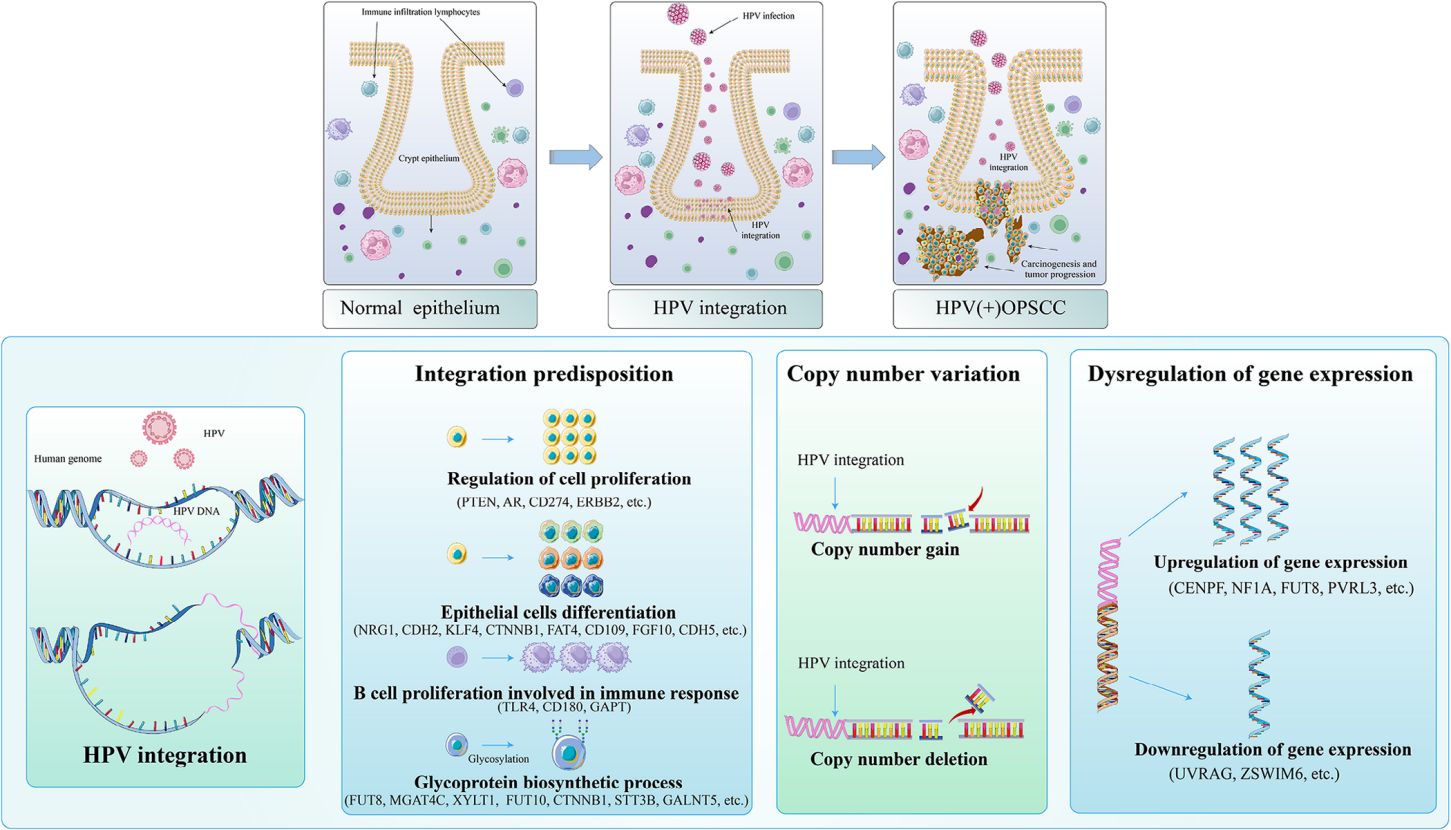
Schematic representation of HPV integration in carcinogenesis. The upper diagram illustrates the progression of oropharyngeal epithelium (mainly crypt epithelium) from initial infection to carcinogenesis. The lower diagram shows the impact of HPV integration during tumour development.

As illustrated in Figure 8, normal oropharyngeal crypt epithelium is infected by HPV initially. After that, the host genome is integrated by HPV gene breakpoints, leading to subsequent malignant transition of normal epithelium. When infected, the host genome is prone to be integrated at specific genes which play important functional roles, such as epithelium differentiation and proliferation, immune cells proliferation, post‐translational modification of proteins such as glycosylation and others. By causing instability of the host genome and subsequent CNVs of the host gene, HPV also facilitates shaping the unique molecular features of HPV(+)OPSCC. The dysregulation of gene expression directly influenced by HPV integration or viral insertion induced CNVs may be a possible mechanism of HPV‐caused tumourigenesis. Collectively, these findings shed new light on the discovery of new mechanisms by which HPV promotes carcinogenesis independent of E6/E7 oncoproteins.

## 4 DISCUSSION

While HPV(+)OPSCC generally responds well to radiotherapy, a subset of patients displays poor response to standard therapy, prompting the need to delve into the underlying mechanisms of HPV‐driven carcinogenesis. In recent years, there has been an increasing focus on investigating HPV integration, with various detection methods such as WGS and RNA‐seq being employed.^14^, ^20^, ^30^ However, the limited sequencing depth in WGS hampers the identification of all single nucleotide integration sites. Conversely, virus–host chimeric transcripts detected by RNA‐seq are usually spliced, and thus may be mis‐mapped from integrant templated.^31^, ^32^ To address this issue, we employed the HIVID technique, known for high sensitivity and depth, to target the viral genome and identify HPV integration sites and their characteristics in OPSCC, encompassing both the host and HPV genome.

It is worth noting that the rate of HPV integration in OPSCC (94.5%) was found to be higher than that in cervical cancer (81.7%) using similar detection methods.^12^ Evidence shows that the rate and number of HPV integration events significantly increase during the transition from cervical intraepithelial neoplasia to cervical cancer, supporting the potential use of HPV integration events as disease progression markers for preventive screening purposes.^12^ One limitation of the study is the lack of non‐tumour tissue as the control. One reason is that the study was focused on the oropharyngeal carcinoma, whereas the non‐tumour tissues infected by HPV have been identified recently only in oral cavity mucosa.^33^ On the other hand, there has been no evidence for the existence of non‐tumour tissue with HPV infection such as precancerous lesions at the oropharynx yet. Therefore, the non‐tumour samples were not included as the control. In future studies, we wish to collect the HPV associated non‐tumour tissue as the control to overcome the limitation. Another limitation of this study was that HIVID may detect some episomal human–virus DNA hybrids. However, as episomal hybrids have been detected in only a minority of HPV(+)OPSCC tumours, and episomal hybrids demonstrated similar structural forms and play similar functional roles compared with intrachromosomal hybrids, we believe episomal status does not influence our conclusion.

The identification of genes with high frequent integration through our study implied the existence of selective advantages for integration in certain genomic loci. Notably, some of these hot spot genes, such as LRP1B, DMD, FHIT, CSMD1, CTNNA3 and DLG2 were located at FSs FRA3B, FRAXC, FRA2K, FRA8B, FRA10D and FRA11F. Given the inherent instability of FSs and their susceptibility to breakage, it is plausible that the instability of these regions facilitates viral integration, leading to consequential genomic alterations. Notably, LRP1B and DLG2 are also integration hot spot genes in cervical cancer identified by long read sequencing.^34^ Moreover, our investigation uncovered instances where HPV integration co‐occurred with gene mutation, notably in the tumour suppressor gene LRP1B. Mutation enrichment of LRP1B has been observed in HPV‐integrated cervical cancer and HPV‐positive HNSCC.^35^ In CC with LRP1B integration, down‐regulation of LRP1B expression was associated with higher expression of total and spliced E6 expression,^12^, ^35^ known to accelerate tumour growth and migration and to increase chemotherapy resistance in other tumours.^36^, ^37^ These evidences supported our hypothesis that fragments of the HPV gene may be more likely to integrate into some specific loci that are prone to genomic alterations, leading to mutations in genes associated with cancer. Additionally, other integration hot spot genes in FSs have been shown to play important roles, such as FHIT, which has been identified as a tumour suppressor. Furthermore, decreased expression of FHIT, DLG2, CTNNA3 and DMD are reported to be associated with disease recurrence.^38^ The important role of FSs in cancer development suggests that HPV integration may increase genomic instability in these regions, contributing to the onset and progression of cancer. CD274, which encodes the immune checkpoint PD‐L1, has been identified as an integration hot spot gene in other studies as well as ours.^13^, ^14^, ^20^ HPV(+)OPSCC has been reported to exhibit higher PD‐L1 expression and immune evasion status than its HPV‐negative counterparts.^18^ Intriguingly, six out of seven samples with HPV‐CD274 integration exhibited elevated PD‐L1 expression (combined positive score > 20). Remarkably, the overall rate of PD‐L1 high expression in HPV(+)OPSCC was reported to be only 29.5% (33 out of 112) in our prior study.^18^ As HPV‐CD274 integration induced PD‐L1 up‐regulation has been reported in several researches,^13^, ^14^, ^20^ we hypothesised HPV integration might be a possible mechanism of high PD‐L1 expression in HPV(+)OPSCC.

Regarding the viral genome, our study demonstrated that HPV DNA could break at any point, but the viral oncogene E6 and E7 are more likely to remain intact. Although a shortened form of E6 can still promote cell proliferation, its function is incomplete and may even inhibit E6‐mediated p53 degradation.^14^, ^39^ Therefore, intact HPV E6 is likely to be preserved, as demonstrated in our study. Intact viral oncogenes are essential for carcinogenesis. For example, intact E2 is necessary for viral–host gene amplification and transcription, while E4 can interact with E2 and aid in E6/E7 viral amplification.^40^ Furthermore, co‐expression of E2 and E4 has been shown to promote cell proliferation in a p53‐dependent manner,^41^ suggesting that intact E2/E4 oncoproteins provide alternative mechanisms of carcinogenesis independent of E6/E7. Our observation that E2, E4, E6 and E7 are likely to remain intact rather than breaking and inserting into the host genome further supports this hypothesis. Our findings are in agreement with Michael Dean and colleagues, and align with the observation that integrated tumours exhibit notably higher viral RNA expression,^16^ which further reinforce our conclusions that tumours featuring elevated viral expression correspond to increased integration numbers.

Unlike HPV(−)HNSCC, which is mostly associated with smoking and exhibits ubiquitous loss of TP53 function,^42^ HPV oncoprotein activation of PI3K–AKT–mTOR1 signalling pathway is the main driver of genomic alterations in HPV(+)OPSCC, including PIK3CA(58%), FGFR3(24%), PTEN(22%) and CYLD(16%), as observed in our study.^3^ The innate immune DNA cytosine deaminase APOBEC family has been shown to restrict viruses,^43^ and E6 has been reported to up‐regulate APOBEC3B expression, which can cause somatic mutations belonging to mutation signatures 2 and 13.^44^ In this study, we observed a positive relationship between TMB and mutation signatures 2 and 13, while the number of HPV integrations was also found to be significantly correlated with signature 13. These findings suggest that virus‐induced anti‐viral innate host response may be an important cause of mutagenesis in HPV(+)OPSCC.

Replication of HPV depends on the up‐regulation of HRR‐genes, such as RAD51 and BRCA1.^45^ Additionally, HPV‐induced activation of ATM DNA damage response facilitates viral genome amplification and viral replication foci formation,^46^ which may explain the reverse association between HPV integration and HRD associated signature 3. HPV can increase HRD by blocking of TGFb signalling,^47^ and down‐regulation of DNA repair caused by HRD may be a mechanism of sensitivity of HPV(+)OPSCC to radiation and platinum‐based anticancer agents.^48^ Based on the reasons discussed above, we hypothesise that HPV integration events may serve as a negative predictor for the radio‐chemotherapy response. Further validation through clinical trials is in necessary.

Peter et al.^49^ reported that in CC, 33% (17/51) of integration sites displayed co‐occurrence of CNV. Additionally, long‐read sequencing of HPV(+)CC SNU‐1000 cell line demonstrated host DNA rearrangement of locus in the vicinity of viral integration sites.^16^ Here, we also observed HPV integration enrichment in loci adjacent to CNV. The recurrence of co‐localisation of viral integration and CNVs at specific sites suggests a selective advantage for viral insertion and subsequent genome change, which plays a functional role in cell differentiation and proliferation. To be noticed, integration sites identified in HPV(+)OPSCC cell line UPCI:SCC090 were also found in UPCI:SCC152, a relapse derived cell line from the same host, suggesting an active role of HPV integration in tumourigenesis rather than a passive role.^16^ Furthermore, Akagi and colleague^31^ proposed a looping model that elucidates the mechanism of HPV integration, suggesting that HPV integration can lead to the formation of HPV‐host circular fusions, resulting in focal amplification and dysregulation of gene expression. In the expanded model utilising long‐read sequencing, Akagi et al.^15^ demonstrated that HPV episome replication induce viral genome instability, facilitating integration into the host genome. The insertion of HPV results in the amplification or recombination of virus–host segments and facilitates the formation of intra‐tumoural heterogeneity and cancer evolution.^15^ Therefore, we believe HPV oncoprotein‐induced host genomic alterations and HPV integration directly cause changes in the genetic structure that cooperate to shape specific molecular spectrum of HPV(+)OPSCC.

Our study demonstrated that genes with HPV integration have significantly different expressions compared with genes without integration, and a total of 59 genes which showed distinct expression value between samples with and without integration were identified. It has been shown that HPV could integrate into transcription factors and act as a super‐enhancer like element, such as Brd4, promoting oncogene transcription.^50^ HPV can also regulate gene expression by directly inserting it into specific genes. For example, inserting upstream of NR4A2 leads to a 248‐fold amplification and elevating of gene expression, integrating with ETS2 results in significant attenuation of its exon 7 and 8, while integration at intron 8 causes significantly up‐regulation of transcripts of RAD51B exons 9−11.^20^ Hence, HPV integration can regulate gene expression through several mechanisms, including integration into transcription factors that regulate oncogene expression, causing gene CNV, direct changes to gene expression levels by integrating into transcription regulation regions and altering adjacent exon levels by integrating into intergenic regions. Here, we observed that genes with integration occurring at introns and promotors were significantly up‐regulated, and most integration sites in genes whose expression was influenced by integration were located at introns and promoters. Therefore, we believe that the latter two mechanisms were the primary ways in which integration regulates gene expression.

We noticed higher frequency of immune‐related genes integrations in subtype of this tumour with abundant TILs, as consistent with the observation in HPV(+)CC that cold immune microenvironment were observed in integration‐negative tumours.^32^ Moreover, integration into immune checkpoints CD274 could potentially contribute to the elevated PD‐L1 expression and immune evasion status. This led us to speculate that viral integration might play a role in shaping the distinct tumour microenvironment of HPV(+)OPSCC. Our investigation unveiled several genes impacted by viral integration, with some potentially involved in carcinogenesis. For instance, high expression of TFDP2 (Transcription Factor Dp‐2), has been documented in HPV(+)HNSCC and HPV(+)CC compared with HPV(−) tumours, highlighting its roles. Concurrence of viral integration, copy number gain and gene up‐regulation further supports potential role of viral insertion.^51^, ^52^ A significant observation was that integrated genes with altered expression were enriched in glycosylation enzymes, including FUT8, GALNT10 and GALNT18. FUT8, the sole enzyme to catalyse core fucosylation, has been implicated in various cancers.^53^ Here, FUT8 was identified as significant integration hot spot genes, with 12.8% samples harbour HPV‐FUT8 integration. High expression of FUT8 was reported to be the main reason for up‐regulation of expression and protein stability of immune checkpoint B7H3, elevating subsequent immunosuppression and suppressing immune response in breast cancer.^54^ Thus, it might serve as a therapeutic target, as fucosylation inhibitor 2F‐Fuc showed synergetic effects with anti‐PD‐L1 therapy.^54^ Also, as shown in Figure S9, a positive correlation between FUT8 and glycosylated PD‐L1 expression can be a possible mechanism of high immune evasion status in HPV(+)OPSCC. GALNT10 and GALNT18 are members of the GalNAc polypeptide N‐acetyl‐galactosaminyltransferases, which catalyse O‐linked glycosylation of mucin and can promote EGFR O‐glycosylation and subsequent AKT phosphorylation, leading to tumour proliferation.^55^, ^56^ Therefore, we speculated that HPV integration may indirectly regulate gene expression linked to post‐translational modifications, thereby contributing to tumourigenesis. CENPF (Centromere Protein F), which displayed distinct expression in samples with viral integration and co‐occurrence of copy number gain, has been identified as a negative prognosis predictor in various cancers, and targeting its farnesylation, in combination with cisplatin, has shown therapeutic promise in nasopharyngeal carcinoma.^57^, ^58^ These examples illustrate how functional HPV integration can bring about in genomic alterations, dysregulation of gene expression, or both, thereby modulating the expression or modification of oncogenes and tumour suppressor genes to drive subsequent carcinogenesis. Therefore, the identification of functional integration site may provide an alternative therapeutic targets for HPV(+)OPSCC.

In this study, by combing HIVID with DNA sequencing and RNA‐seq, we demonstrated that HPV could drive tumourigenesis by inducing alterations in host genomic and transcriptomic profiles at integration sites, suggesting that targeting integration sites might be a promising treatment strategy. Unlike cervical cancer, which benefits from established precancerous lesion identification and screening strategies, the absence of such strategies for oropharyngeal cancers presents a challenge. In the context of cervical cancer, ongoing clinical trials are investigating the significance of HPV integration status in screening approaches,^59^ offering valuable insightful for potential screening strategies in HPV(+)OPSCC. Additionally, noteworthy progress has been made in gene editing therapies targeting HPV oncogene E6 and E7 in Hela cells, leading to the up‐regulation of tumour suppressor proteins p53/Rb.^60^ Therefore, gene editing technologies hold promise for targeting genomic sites with viral integration or cells harbouring viral insertions. As highlighted earlier, HPV integration may lead to dysregulation of oncogenes, exerting carcinogenic effects independently of E6 and E7. Hence, strategies aimed at targeting viral‐integration induced oncoproteins offer therapeutic potential for HPV(+)OPSCC. For instance, in cervical cancer, the integration of HPV in the CCDC106 gene resulted in its overexpression and subsequent tumour progression by interacting with tumour suppressor p53 and causing p53 degradation, suggesting its use as a potential therapeutic target.^61^, ^62^, ^63^ The development of therapeutic vaccines based on antigens presented by HPV oncoproteins, whose genes have integrated into the human genome, presents another avenue for treatment. Furthermore, personalised therapeutic vaccines based on virus–host proteins offer a tailored approach treating patients with HPV(+)OPSCC where viral integration has occurred.

In conclusion, our study provides compelling evidence that HPV integration is a prevalent event in HPV(+)OPSCC. Our findings confirm the existence of integration hot spot genes and a preference for viral insertion into specific genomic regions within the human genome. We also identified unstable breakpoints within the HPV genome, hinting at selective advantages during integration. Notably, tumours arising from tonsil or oropharyngeal wall and tumours displaying elevated PD‐L1 expression exhibit a higher frequency of integration sites, suggesting an interplay between the tumour microenvironment and HPV integration. We also observed an enrichment of viral integration in regions of the host genome characterised by CNVs, suggesting a dynamic interplay between HPV fragments and the host genome that contributes to the unique molecular landscape of HPV(+)OPSCC. Moreover, genes affected by viral integration demonstrated distinct expression patterns compared with non‐integrated genes, and integrated genes play crucial roles in cellular functions. Overall, we investigated the characteristics of HPV integration in OPSCC by mapping HPV breakpoints on the human host genome in a large sample cohort and characterised the interactive effects of HPV integration on the host genome. Additionally, our study showed that genomic alterations and dysregulation of gene expression level induced by HPV integration may be a potential tumourigenesis mechanism independent of E6 and E7 oncoproteins, while targeting integration sites could hold promise as an innovative treatment strategy.

## AUTHOR CONTRIBUTIONS

*Conception and design*: Shengming Xu, Jiang Li, Zhiyuan Zhang and Lizhen Wang. *Developmental of methodology*: Shengming Xu and Jiang Li. *Acquisition of data*: Chaoji Shi, Rong Zhou and Shuli Liu. *Analysis and interpretation of data*: Shengming Xu, Niannian Li, Yong Han, Chuxiang Qu Ronghui Xia, Chunye Zhang and Yuhua Hu. *Writing, review and/or revision of the manuscript*: Shengming Xu, Jiang Li and Zhen Tian. *Administrative, technical or material support*: Jiang Li, Zhiyuan Zhang, Lizhen Wang and Zhen Tian. *Study supervision*: Jiang Li, Zhiyuan Zhang and Lizhen Wang.

## CONFLICT OF INTEREST STATEMENT

The authors declare no potential conflict of interest.

## ETHICS STATEMENTS

The study was approved by the Ethics Committee of Shanghai Ninth People's Hospital (approval number: SH9H‐2022‐T230‐2). Written, informed consent for participation was not required for this study in accordance with the national legislation and the institutional requirements.

## Supporting information

Supporting Information

Supporting Information

Supporting Information

Supporting Information

Supporting Information

Supporting Information

Supporting Information

Supporting Information

Supporting Information

Supporting Information

Detailed information of each sample.

Gene list of the cancer panel containing 605 cancer‐related genes

HPV integrtion sites detected by HIVID

Integration sites validated by Sanger sequencing

Number of breakpoints in each viral gene

## Data Availability

The data generated in this study are available within the article and its supplementary data files. All data supporting the findings of this study are available from the corresponding authors upon. reasonable request.
