# Supplementary material for: Mapping the landscape of HPV integration and characterising virus and host genome interactions in HPV‐positive oropharyngeal squamous cell carcinoma
Source: Clin Transl Med. 2024 Jan 27;14(1):e1556. doi: 10.1002/ctm2.1556 (PMC10819103; doi:10.1002/ctm2.1556)
Supplement: Supplementary file 1 — Supporting Information [file CTM2-14-e1556-s012.docx]

**Supplementary Figure 1.** Validation of integration sites. A. Representative image of HPV(+)OPSCC tested by HPV RNA in situ hybridization. The brown dots indicate HPV  **E6/E7 transcripts** which are detected in tumor cells. B. The workflow of HIVID. C. PCR validation of randomly chosen integration sites V1-V23. D. Consistency between viral insertion breakpoints detected by WES and HIVID. Blue: number of consistent viral breakpoints; Yellow: number of inconsistent viral breakpoints. E. Distribution of consistent HPV integration sites detected by HIVID and WES in Sample S108. The color key indicates number of aligned reads. F. Consistency between viral insertion breakpoints detected by RNA-sequencing and HIVID. Blue: number of consistent viral breakpoints; Yellow: number of inconsistent viral breakpoints. G. Distribution of consistent HPV integration sites detected by HIVID and RNA-sequencing in Sample S110. The color key indicates number of consistent reads.

**Supplementary Figure 2**. Immunohistochemistry staining images of PD-L1 expression in selected samples with HPV-CD274 integration, including sample S009 (A), sample S011 (B), sample S033 (B), sample S090 (D), sample S100 (E), and sample S112 (F).

**Supplementary Figure 3.** Distribution of HPV breakpoints in various HPV genome. A. Distribution of HPV breakpoints in the HPV 16 genome in 3 samples (genotype of S057, S061, and S078 is NC001526.4). B. Distribution of HPV breakpoints in the HPV 18 genome in 5 samples (HPV 18, genotype of S085 and S095 is AY262282.1; genotype of S038 is GQ180784.1; genotype of S032 and S097 is GQ180789.1 ). C. Distribution of HPV breakpoints in the HPV 33 genome in sample S037 (genotype: HQ537691.1). D. Distribution of HPV breakpoints in the HPV 35 genome in sample S024 (genotype: HQ537713.1). The graphs illustrate the positions of HPV breakpoints within each specific HPV genotype.

**Supplementary Figure 4.** A. Fraction (y-axis) of single nucleotide variants (SNVs) (x-axis) in all 96 possible mutation sequence contexts identified in each of the 67 HPV(+)OPSCC samples. B. Correlations between the proportion of mutation signature 2 and tumor mutation burden (TMB) in the 67 samples. C. Correlations between the proportion of mutation signature 13 and TMB in the 67 samples. D. Correlations of proportion of mutation signature 13 and the numbers of HPV integration sites across the 67 samples. E. Correlations of proportion of mutation signature 3 and the numbers of HPV integration sites in the 67 samples.

**Supplementary Figure 5.** Focal gain (A) and deletion (B) of copy number alterations in 29 samples revealed by GISTIC analysis. Genes exhibiting co-occurrence of copy number variations (CNVs) and HPV integration are displayed.

**Supplementary Figure 6.** A. Distribution of copy number of integrated genes co-located with nearby CNVs from HPV(+)OPSCC samples of this study and the same gene from 39 HPV(-)HNSCC samples randomly chosen from TCGA database. Red dots represent copy number of genes from HPV(-)HNSCC. Blue dots represent copy number of integrated genes from HPV(+)OPSCC. B. Quantile-quantile (Q-Q) plot confirms differences between copy number of integrated genes co-located with CNVs from HPV(+)OPSCC samples in this study (y-axis) and the same genes in 39 HPV(-)HNSCC samples (x-axis) derived from TCGA database (P < 2.2e-16, Kolmogorov-Smirnov test). C. Circos plot illustrating the relationship between the HPV genome and human genes with co-occurrence of viral integration and CNVs in the sample S094. D. Circos plot showing connection between the HPV genome and the human genes with co-occurrence of viral integration and CNVs in the sample S102. The inner circle depicts a histogram with units representing log_2_(copy number)-1. The red squares indicate the position of copy number gained genes with viral integration. The blue triangle indicate the position of deleted genes with viral integration. The lines of different colors indicate different HPV genes.

**Supplementary Figure 7.** Analysis of Tumor infiltrating lymphocytes (TILs) expression analysis. A. Classification of samples into “cold immune”(labeled as green on top) and “hot immune” (labeled as red on top) groups based on TILs expression. B. Representative image of cold immune tumor. C. Representative image of hot immune tumor. D. Comparison of the numbers of CD68 positive TILs in stroma regions of tumors. E. Decreased CD68-positive TILs infiltration in stromal regions of tumors classified as “cold immune” group. F. Increased CD68-positive TILs infiltration in stromal regions of tumors classified as “hot immune” group. G. Comparison of the numbers of CD8 positive TILs in tumor stroma regions. H. Reduced infiltration of CD8-positive TILs in stromal regions of tumors classified as "cold immune" group. I. Enhanced infiltration of CD8-positive TILs in stromal regions of tumors classified as "hot immune" group. J. Comparison of the numbers of CD4-positive TILs. K. Lower infiltration of CD4-positive TILs in tumors classified as "cold immune" group. L. Elevated infiltration of CD4-positive TILs in tumors classified as "hot immune" group. CD20-positive cells were stained as yellow; CD68-positive cells were stained as green; CD4-positive cells were stained as red; Foxp3-positive cells were stained as orange; CD8-positive cells were stained as cyan; P16-positive cells were stained as purple; DAPI for nuclei.

**Supplementary Figure 8.** A. Quantile-quantile (Q-Q) plot confirms differences between expression of integrated genes with dysregulated expression from HPV(+)OPSCC (y-axis) and expression of the same genes from 40 HPV(-)HNSCC (x-axis) derived from TCGA database (P < 2.2e-16, Kolmogorov–Smirnov test). B. Gene ontology analysis of genes with HPV integration and significant outlier expression.

**Supplementary Figure 9.** A. Positive correlations between FUT8 and CD274 RNA expression levels. B. Positive correlations between FUT8 and glycosylated PD-L1 expression levels as determined by immunohistochemistry staining. Representative images of FUT8 (C) and glycosylated PD-L1 (clone:22C3) (D) immunohistochemistry staining in the same sample with high expression of these two genes.
