# Supplementary figures and images for: Mapping the landscape of HPV integration and characterising virus and host genome interactions in HPV‐positive oropharyngeal squamous cell carcinoma
Source: Clin Transl Med. 2024 Jan 27;14(1):e1556. doi: 10.1002/ctm2.1556 (PMC10819103; doi:10.1002/ctm2.1556)

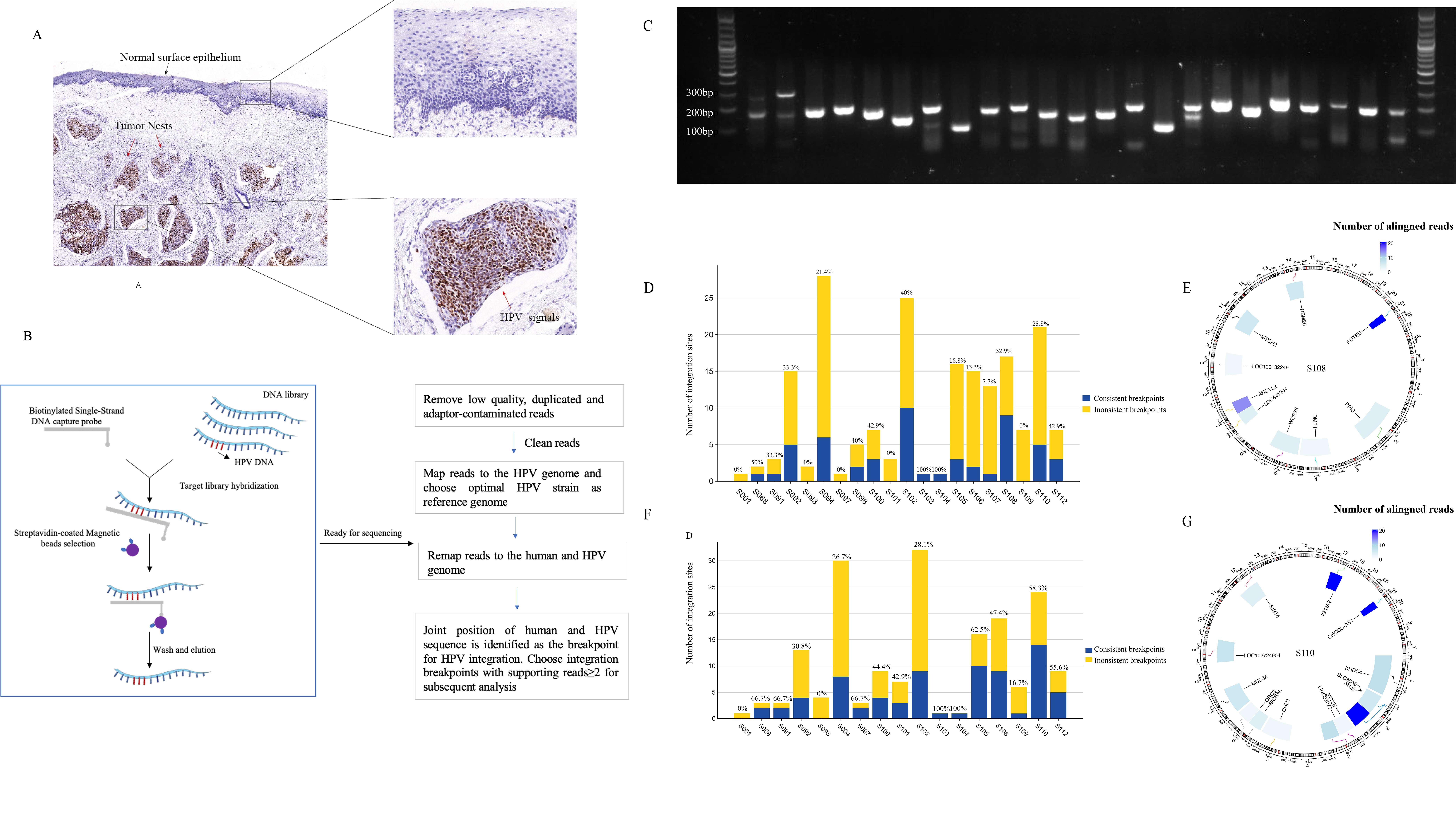

Supplement: Supplementary file 2 — Supporting Information [file CTM2-14-e1556-s007.jpg]

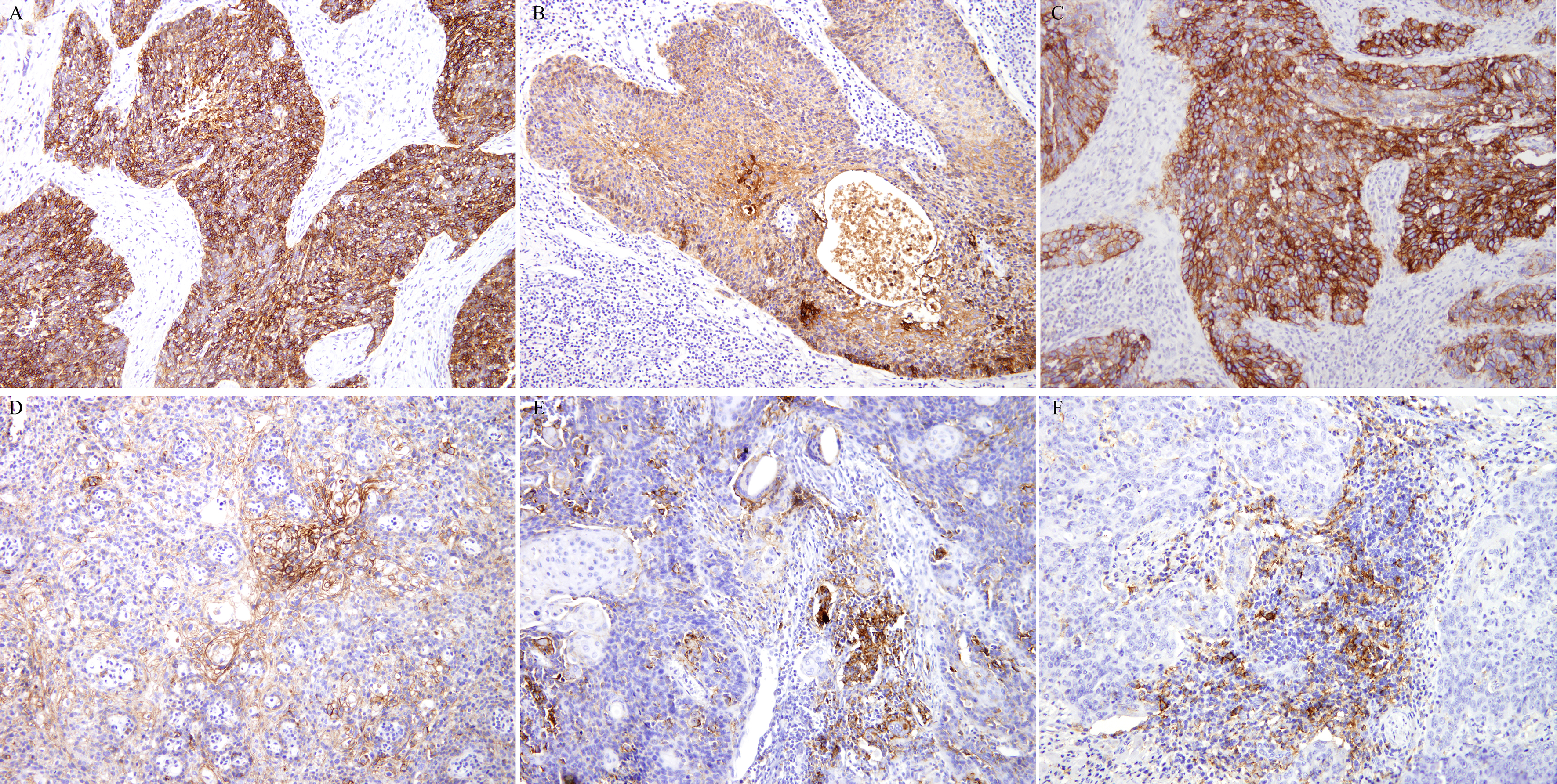

Supplement: Supplementary file 3 — Supporting Information [file CTM2-14-e1556-s008.jpg]

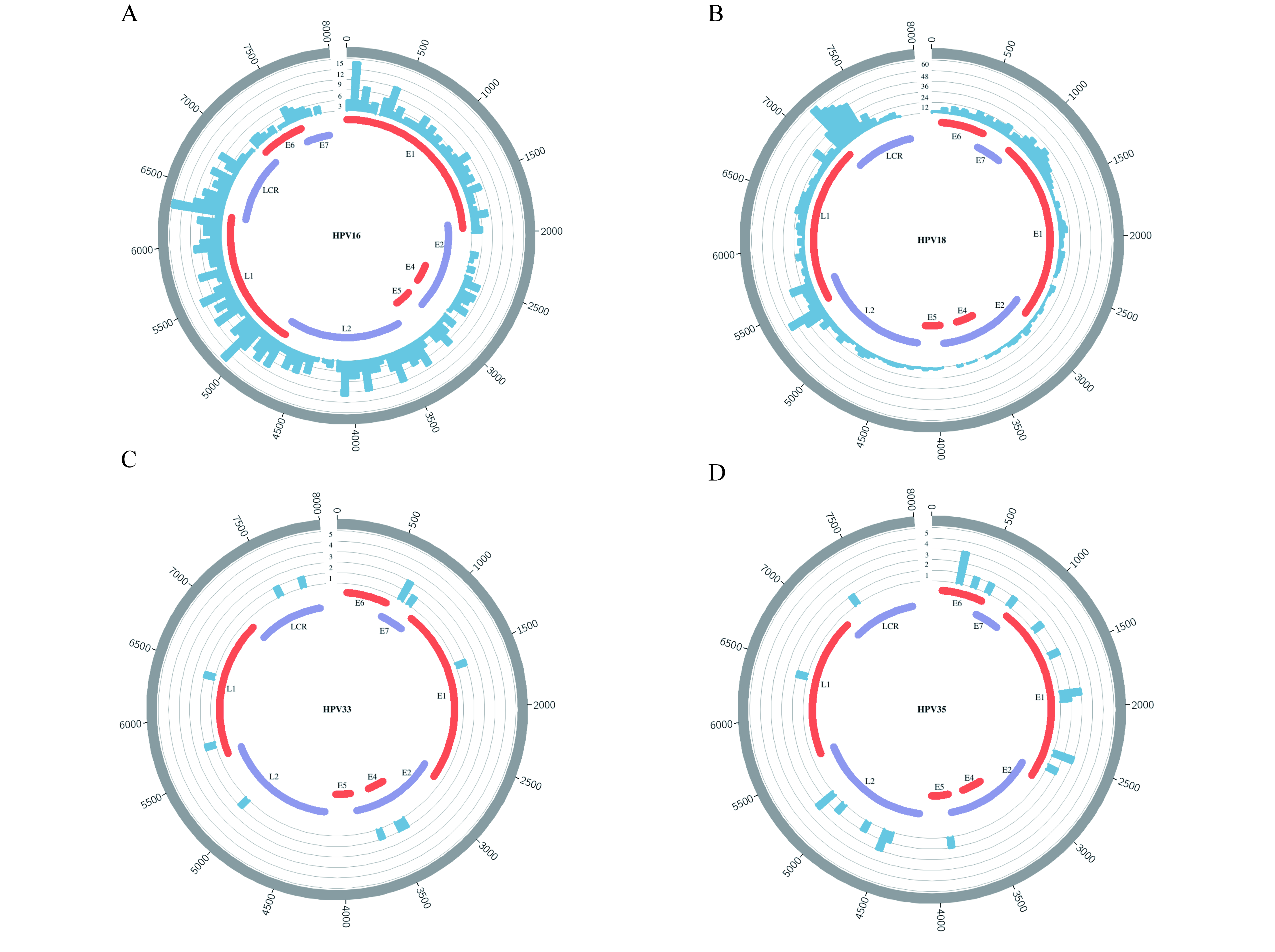

Supplement: Supplementary file 4 — Supporting Information [file CTM2-14-e1556-s001.tif]

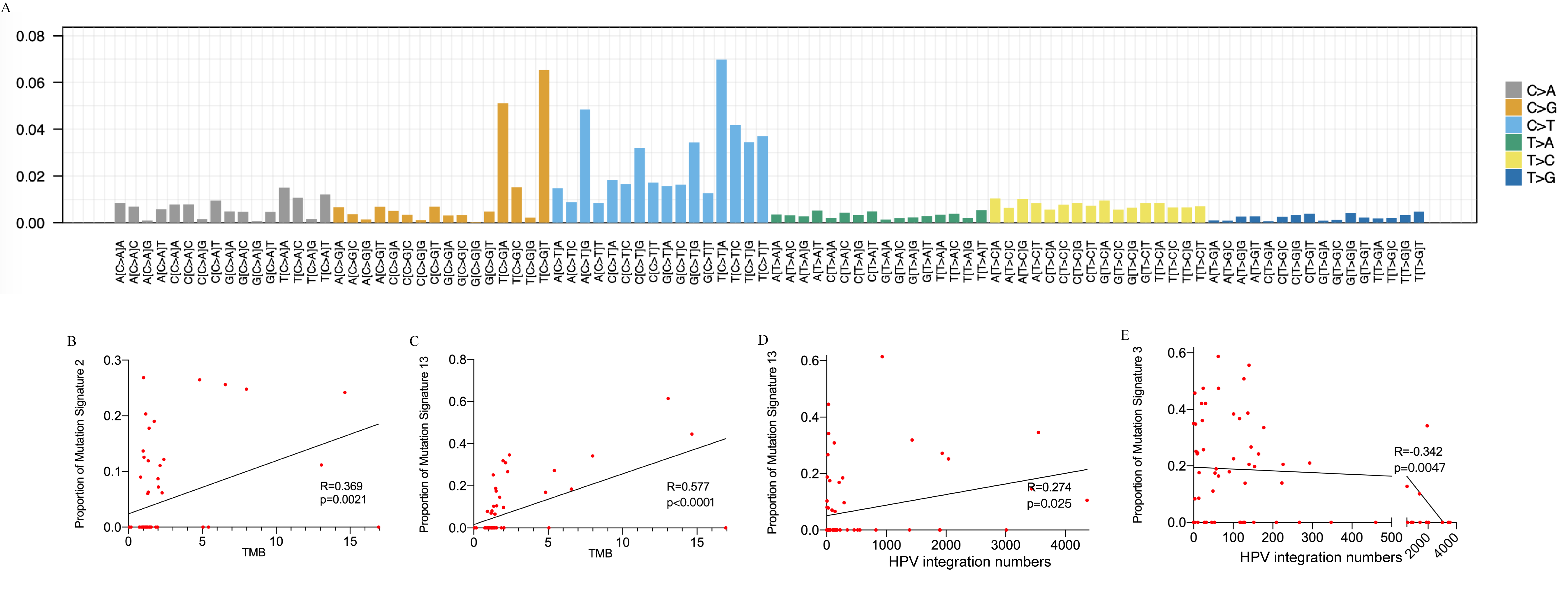

Supplement: Supplementary file 5 — Supporting Information [file CTM2-14-e1556-s004.tif]

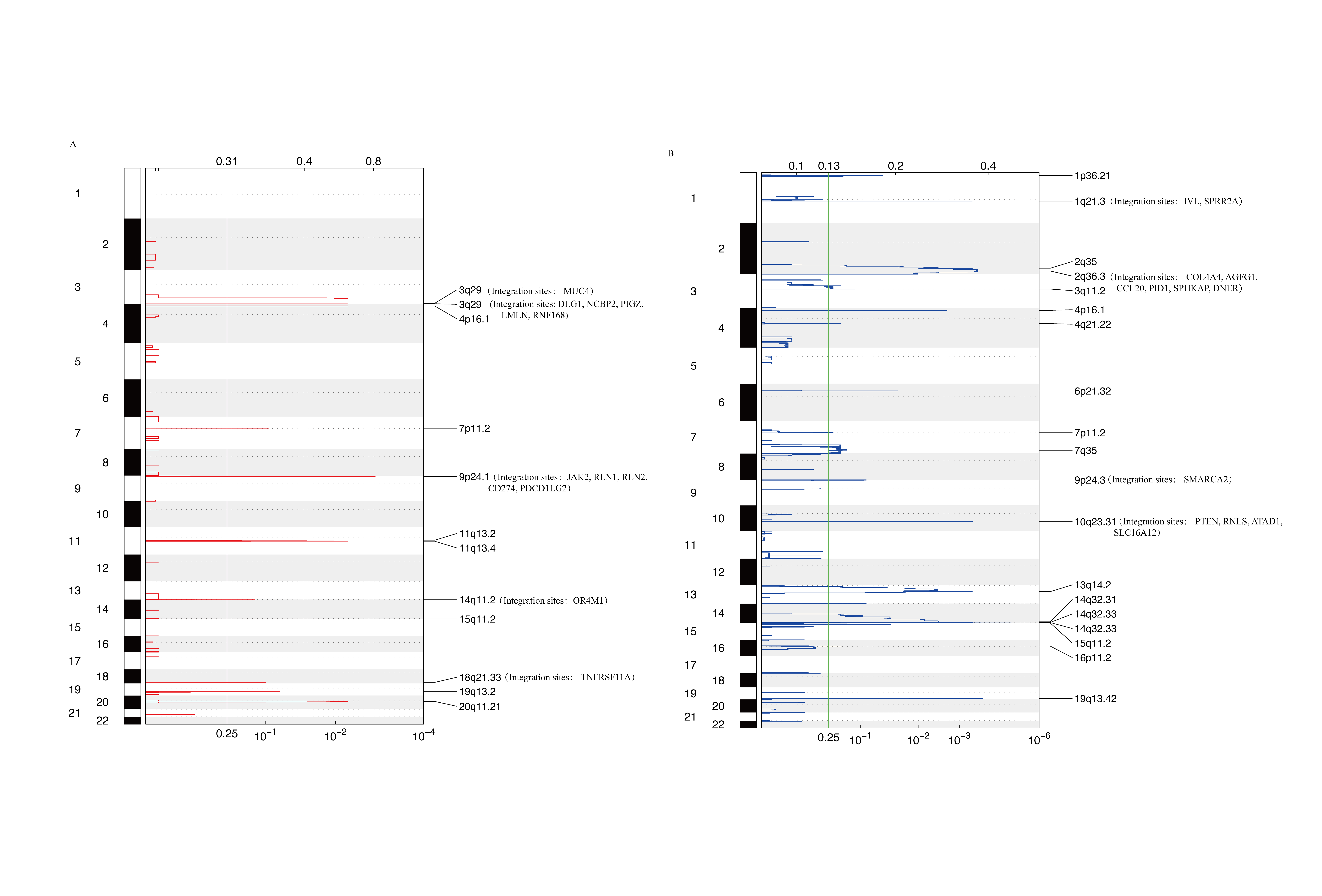

Supplement: Supplementary file 6 — Supporting Information [file CTM2-14-e1556-s006.tif]

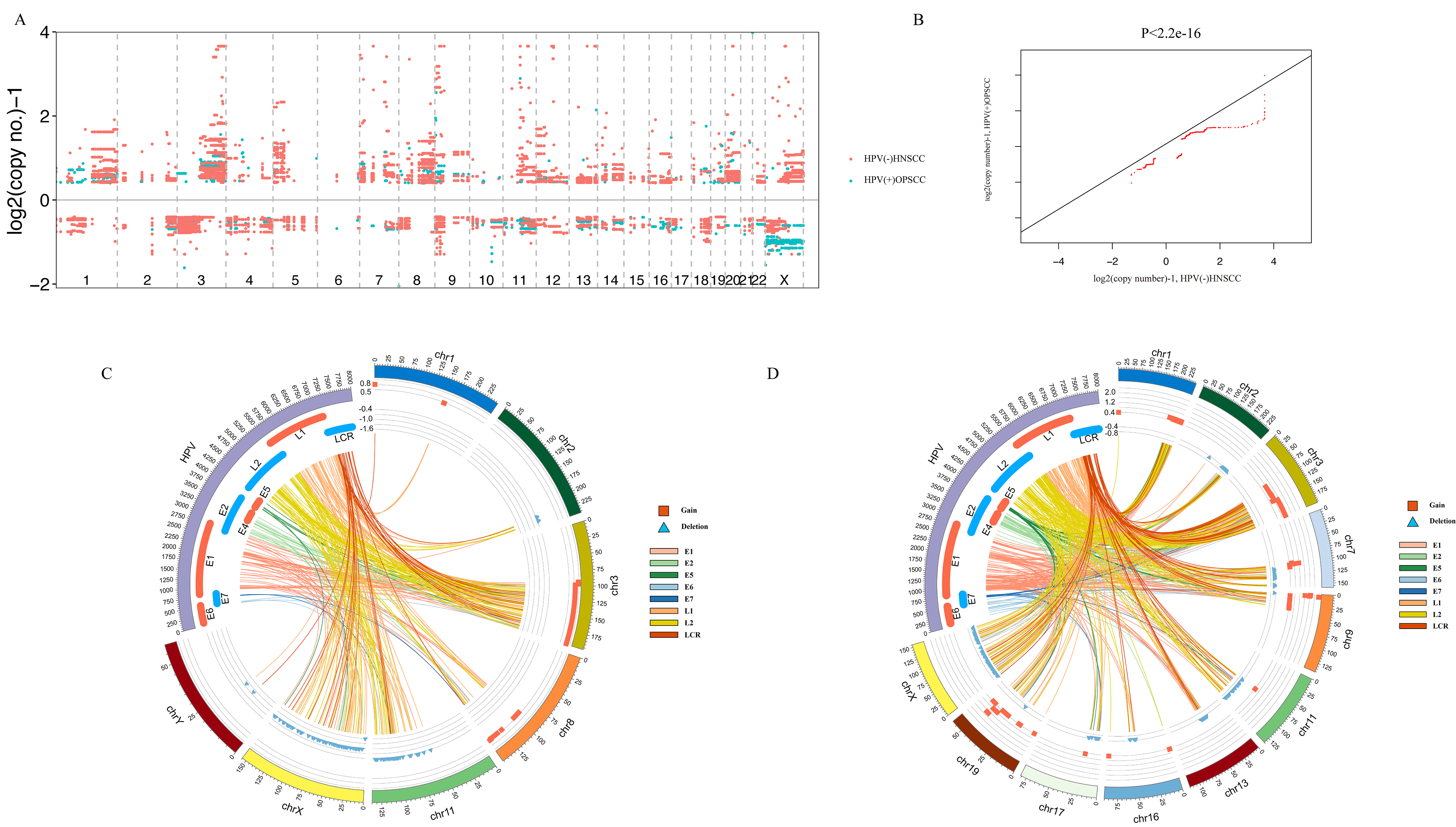

Supplement: Supplementary file 7 — Supporting Information [file CTM2-14-e1556-s010.tif]

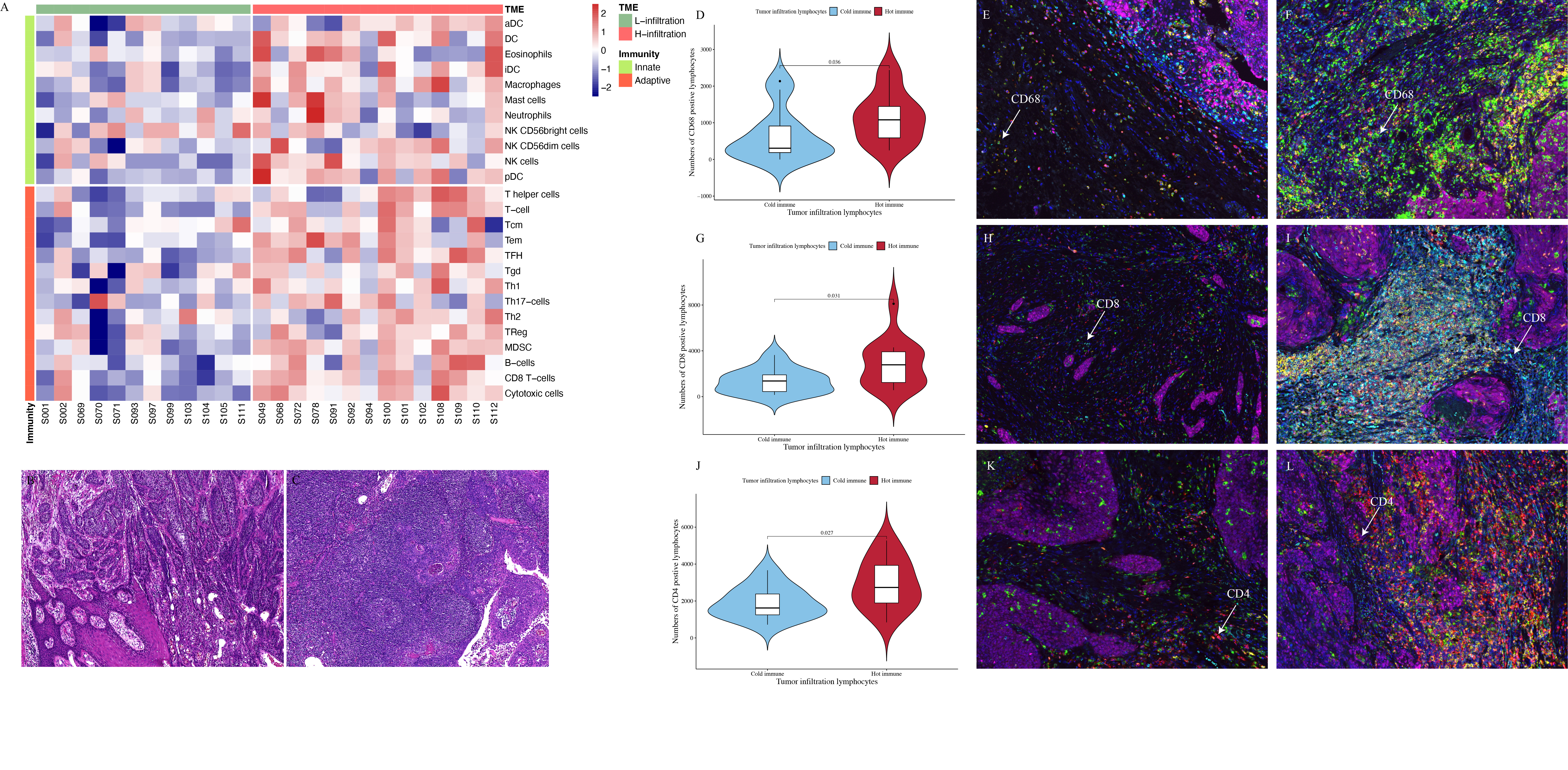

Supplement: Supplementary file 8 — Supporting Information [file CTM2-14-e1556-s003.jpg]

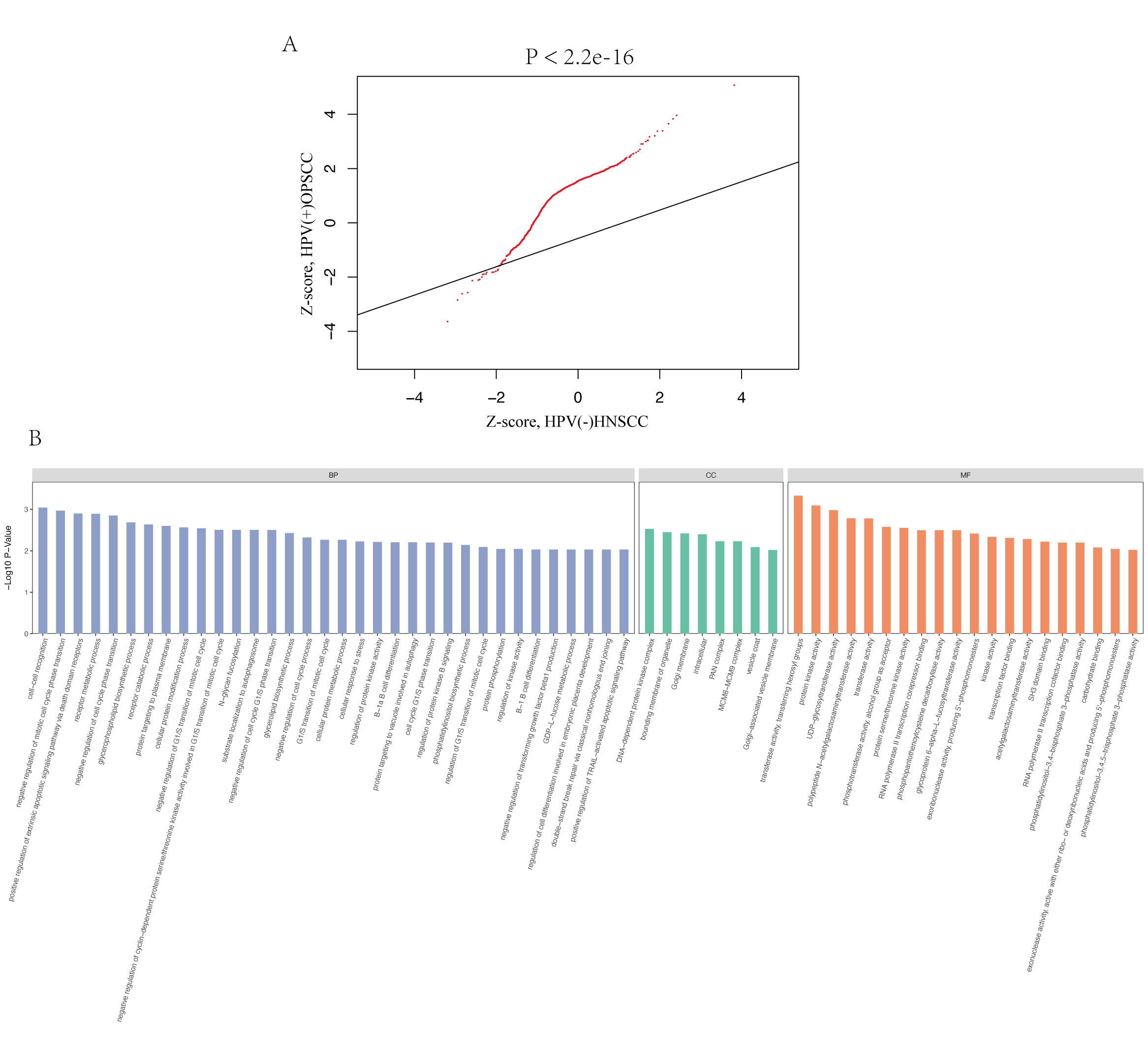

Supplement: Supplementary file 9 — Supporting Information [file CTM2-14-e1556-s014.tif]

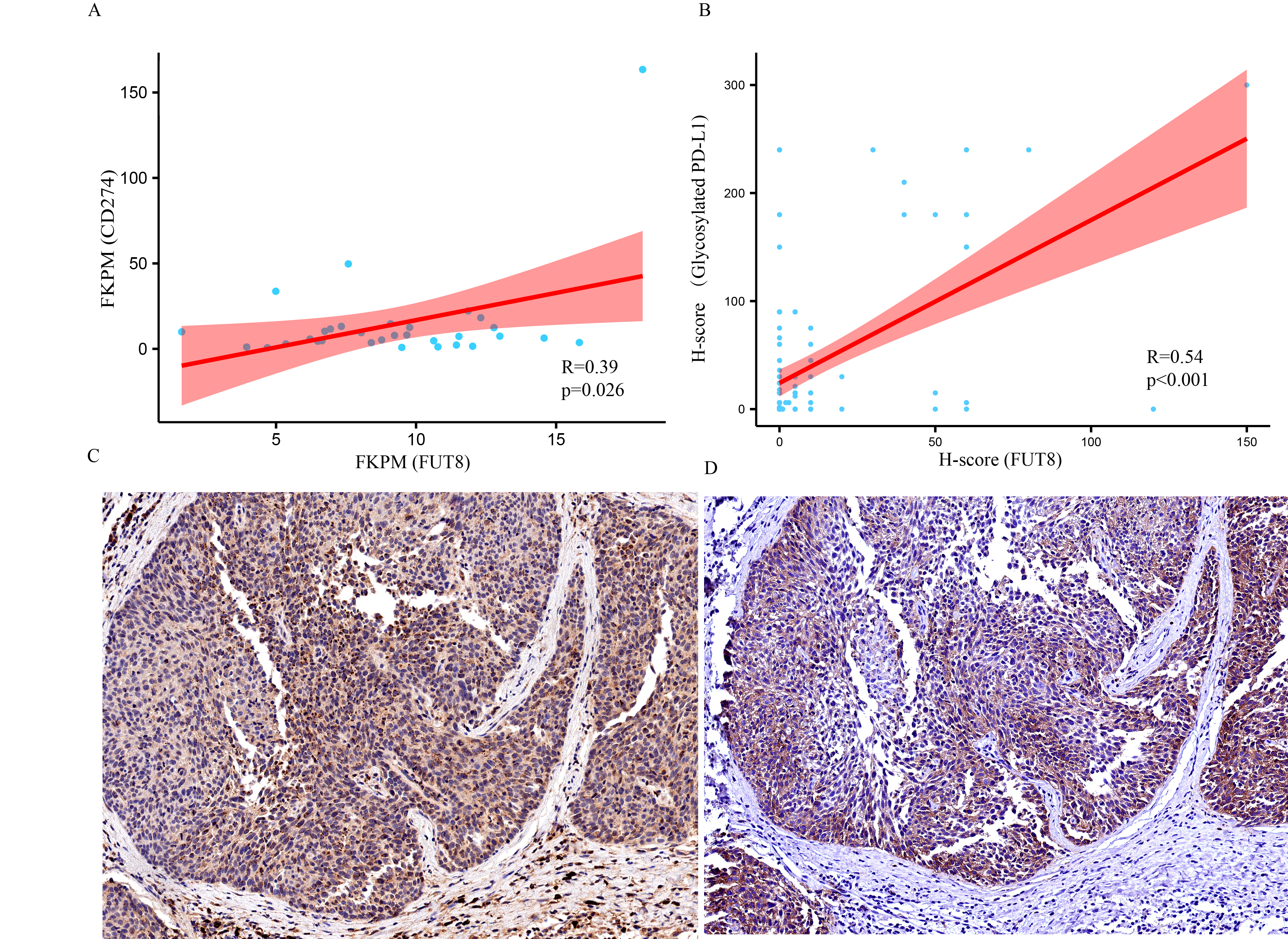

Supplement: Supplementary file 10 — Supporting Information [file CTM2-14-e1556-s005.jpg]
